# Supplementary material for: Subthalamic stimulation modulates context-dependent effects of beta bursts during fine motor control
Source: Nat Commun. 2024 Apr 12;15:3166. doi: 10.1038/s41467-024-47555-3 (PMC11009405; doi:10.1038/s41467-024-47555-3)
Supplement: Supplementary file 1 — Supplementary Information [file 41467_2024_47555_MOESM1_ESM.pdf]

Subthalamic stimulation modulates context-dependent effects of beta bursts during fine motor control

Supplementary figures and tables

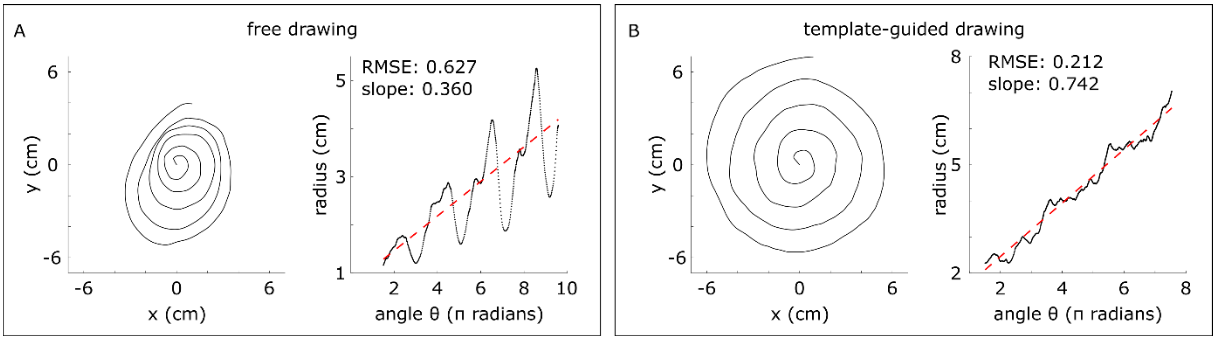

Supplementary Figure 1. Quantification of spatial characteristics. To quantify spatial characteristics of the drawing we performed a radius-angle-transformation,[1] fitted linear models and calculated the root mean square error (RMSE) and the slope of the regression line (red dashed line) as parameters of deviation from an optimal spiral. **(A)** Example of free drawing. **(B)** Example of template-guided drawing.

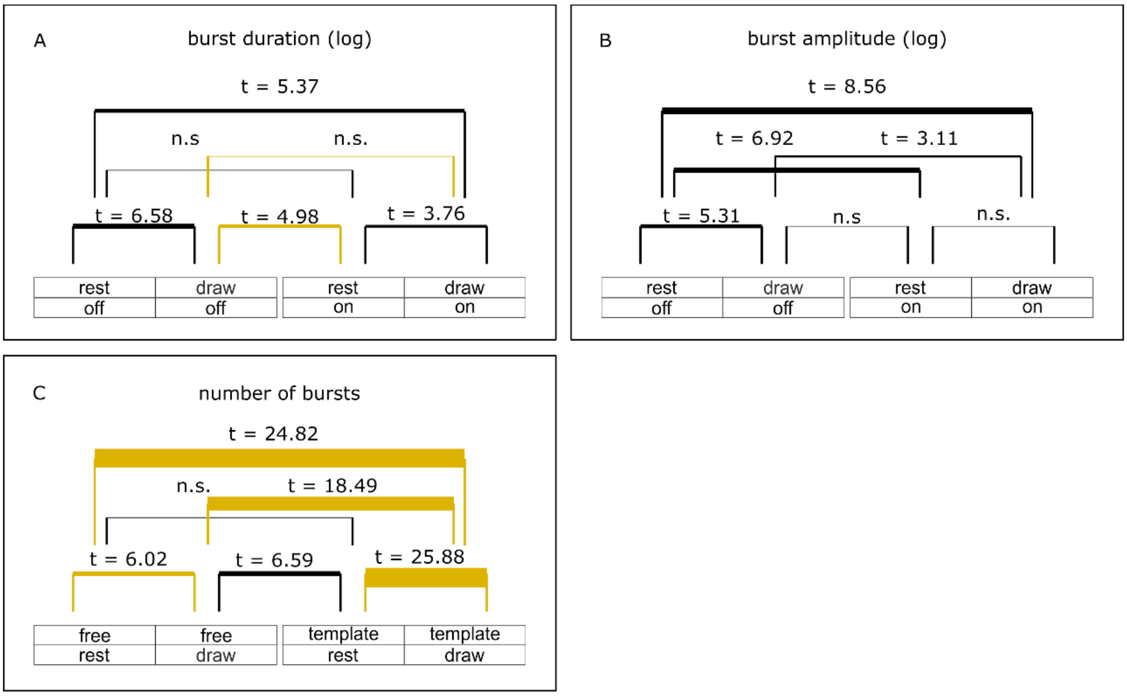

Supplementary Figure 2. Effects of stimulation, movement interval, and drawing condition on beta burst characteristics: post hoc tests. (A) Pairwise estimated marginal means post hoc tests for beta burst duration (rest_off n = 336, draw_off n = 306, rest_on n = 329, draw_on n = 320). **(B)** Pairwise estimated marginal means post hoc tests for beta burst amplitude (rest_off n = 336, draw_off n = 305, rest_on n = 329, draw_on n = 320). **(C)** Pairwise estimated marginal means post hoc tests for number of beta bursts (free_rest n = 320, free_draw n = 302, template_rest n = 345, template_draw n = 324). T-score represents the difference between the condition belonging to the left vertical bar minus the condition belonging to the right vertical bar. Line thickness represents the t-score, while the color codes the direction of the effect (black: left > right; gold: right > left). Detailed statistics are provided in Supplementary Table 3.

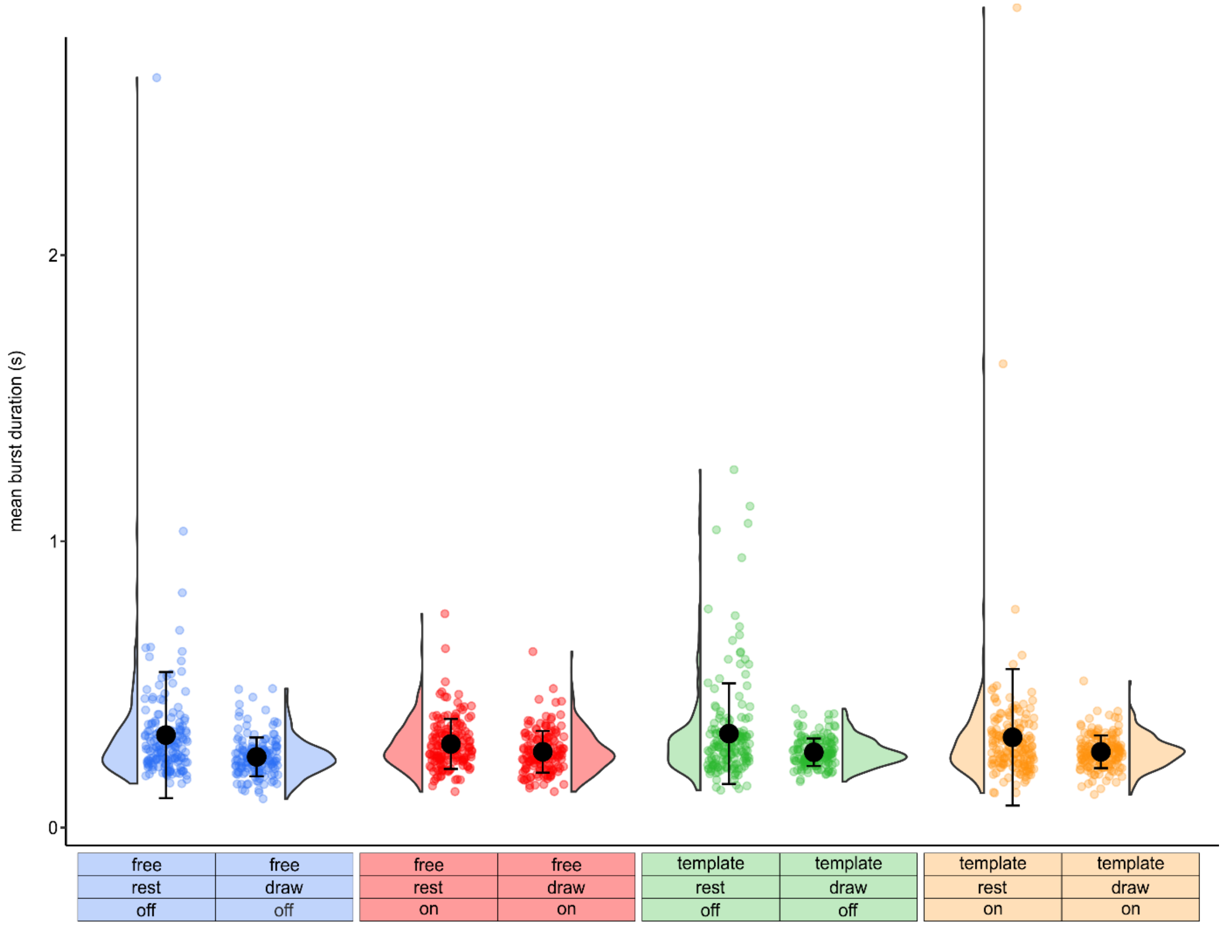

Supplementary Figure 3. Burst duration is reduced during drawing in comparison to the rest interval. Single trials, distributions, mean and standard deviation of the mean burst duration are plotted for the different conditions and intervals (blue = free drawing without stimulation, draw: 0.246 s ± 0.068 (mean ± standard deviation), *n* = 149, rest: 0.323 s ± 0.220, *n* = 164; red = free drawing with stimulation, draw: 0.264 s ± 0.073, *n* = 153, rest: 0.291 s ± 0.087, *n* = 156; green = template-guided drawing without stimulation, draw: 0.263 s ± 0.048, *n* = 157, rest: 0.328 s ± 0.176, *n* = 172; orange = template-guided drawing with stimulation, draw: 0.264 s ± 0.057, *n* = 167, rest: 0.315 s ± 0.238, *n* = 173). Two-sided linear mixed-effects models showed a significant effect of movement interval ($P < 0.001$).

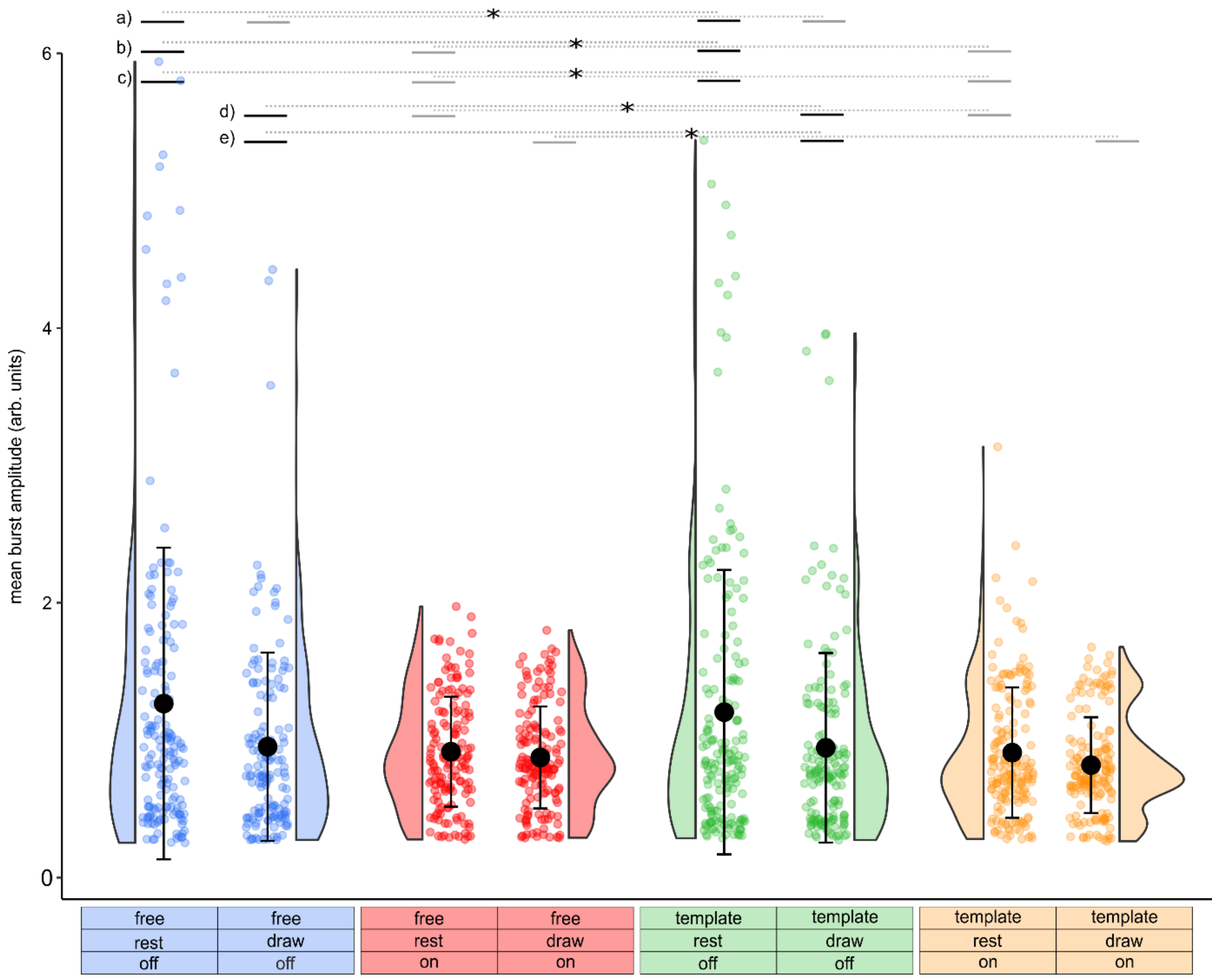

Supplementary Figure 4. Burst amplitude is reduced during drawing in comparison to the rest interval and reduced by deep brain stimulation. Single trials, distributions, mean and standard deviation of the mean burst amplitude are plotted for the different conditions and intervals (blue = free drawing without stimulation, draw: 0.952 arbitrary units (arb. unit) ± 0.686 (mean ± standard deviation, n = 149), rest: 1.266 arb. unit ± 1.134, n = 164; red = free drawing with stimulation, draw: 0.874 arb. unit ± 0.371, n = 153, rest: 0.916 arb. unit ± 0.401, n = 156; green = template-guided drawing without stimulation, draw: 0.944 arb. unit ± 0.690, n = 157, rest: 1.204 arb. unit ± 1.036, n = 172; orange = template-guided drawing with stimulation, draw: 0.818 arb. unit ± 0.348, n = 167, rest: 0.909 arb. unit ± 0.474, n = 173). Two-sided linear mixed-effects models showed significant effects of movement interval (P < 0.001), stimulation (P < 0.001), and a significant interaction between stimulation and movement interval (P = 0.008). The top lines indicate the results from the significant post hoc tests (two-sided, Bonferroni corrected), combined across the drawing conditions (free and template): a) rest_off > draw_off, P < 0.001; b) rest_off > rest_on, P < 0.001; c) rest_off > draw_on, P < 0.001; d) rest_on < draw_off, P < 0.001; e) draw_off > draw_on, P < 0.001. * P < 0.05 (Bonferroni corrected).

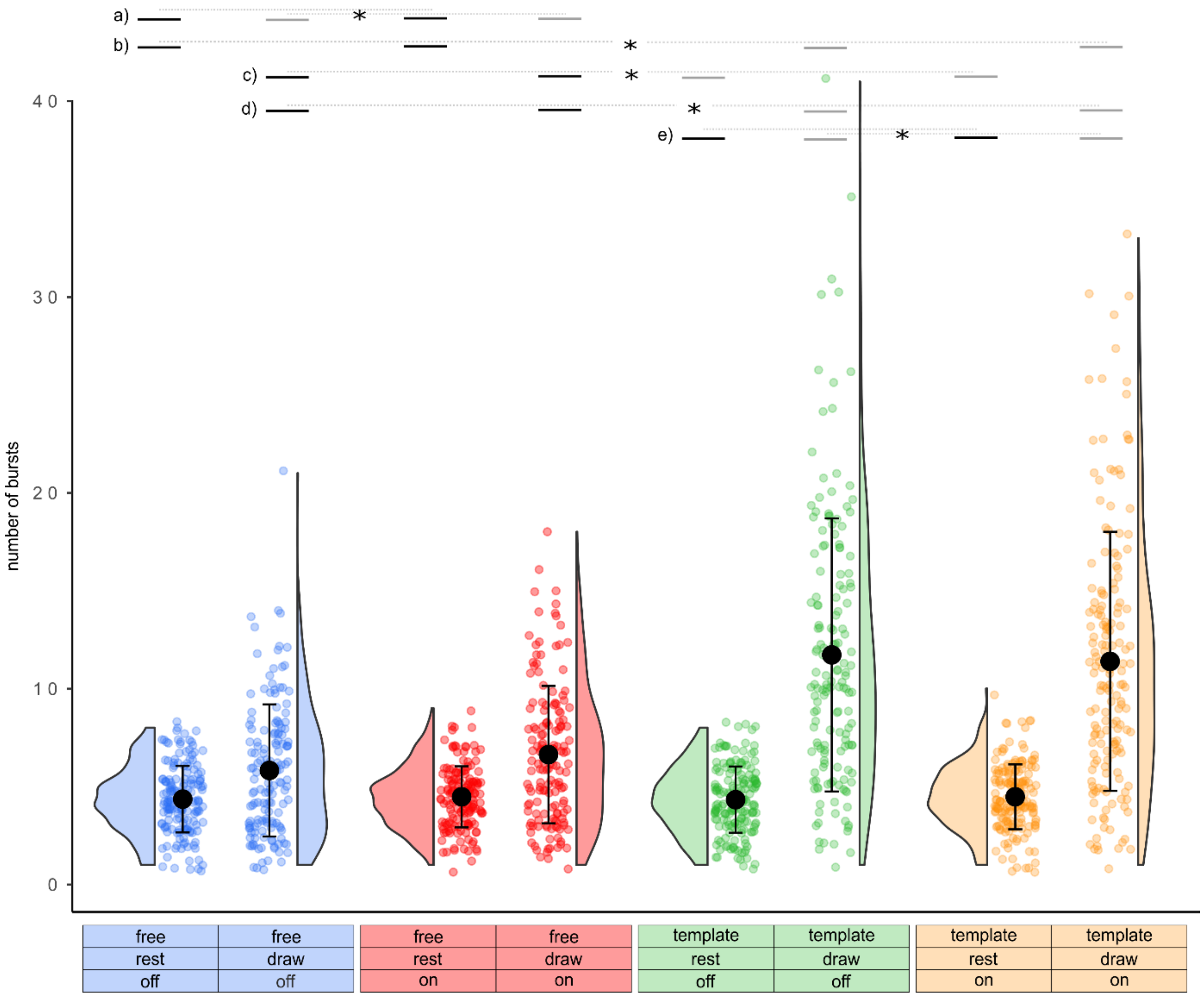

Supplementary Figure 5. Number of bursts. Single trials, distributions, mean, and standard deviation of the number of bursts are plotted for the different conditions and intervals (blue = free drawing without stimulation, draw: 5.819 ± 3.373 (mean ± standard deviation) , *n* = 149, rest: 4.354 ± 1.697, *n* = 164; red = free drawing with stimulation, draw: 6.627 ± 3.505, *n* = 153 rest: 4.474 ± 1.559, *n* = 156; green = template-guided drawing without stimulation, draw: 11.720 ± 6.973, *n* = 157, rest: 4.326 ± 1.692, *n* = 172; orange = template-guided drawing with stimulation, draw: 11.389 ± 6.615, *n* = 167, rest: 4.474 ± 1.655, *n* = 173). Two-sided linear mixed-effects models showed a significant effect of movement interval ($P = 0.002$) as well as an interaction between drawing condition and movement interval ($P < 0.001$). The top lines indicate the results from the significant post hoc tests (two-sided, Bonferroni corrected), combined across the stimulation conditions (on and off): a) free_rest < free_draw, $P < 0.001$; b) free_rest < template_draw, $P < 0.001$; c) free_draw > template_rest, $P < 0.001$; d) free_draw < template_draw, $P < 0.001$; e) template_rest < template_draw, $P < 0.001$. * $P < 0.05$ (Bonferroni corrected).

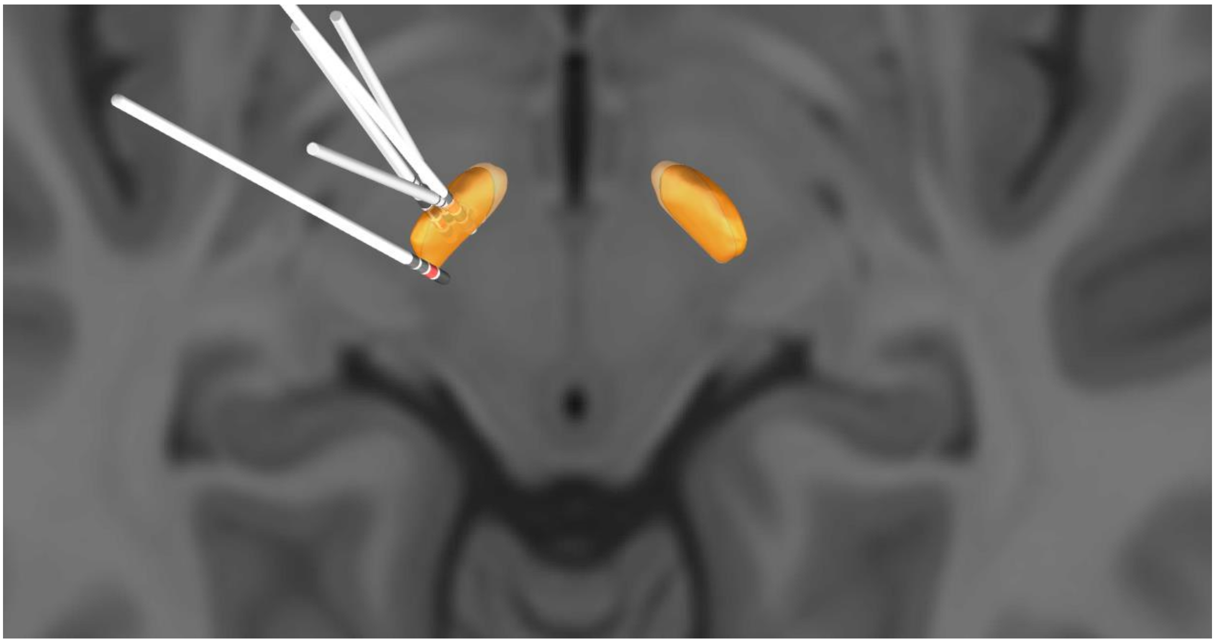

Supplementary Figure 6. Electrode locations of patients with minor or no clinical improvement. Five participants (patients 5, 6, 10, 14, and 18) showed only minor or no clinical improvement of total $UPDRS_{III}$ when receiving DBS during the study. To test if stimulation yielded subclinical effects on drawing execution, we performed two-sided linear models with velocity and RMSE as dependent variables and stimulation and drawing conditions as independent variables for each of these subjects individually. Congruent with our behavioral findings when analyzing the whole cohort, patients 14 and 18 showed increased drawing velocity ($P < 0.001$, $n = 40$, and $P = 0.002$, $n = 40$, respectively), an increased RMSE ($P < 0.001$, $n = 38$, and $P = 0.003$, $n = 40$, respectively), and an increased slope ($P < 0.001$, $n = 40$, and $P = 0.019$, $n = 40$, respectively) when being stimulated. In patients 6 and 10 we did not find any effects of stimulation on velocity ($P = 0.403$, $n = 40$ and $P = 0.131$, $n = 22$, respectively) or RMSE ($P = 0.127$, $n = 16$ and $P = 0.728$, $n = 12$, respectively). DBS slowed down drawing speed in patient 5 ($P < 0.001$, $n = 32$), while decreasing RMSE ($P < 0.001$, $n = 32$). In this patient, the electrode was positioned slightly outside the left STN (left orange region).

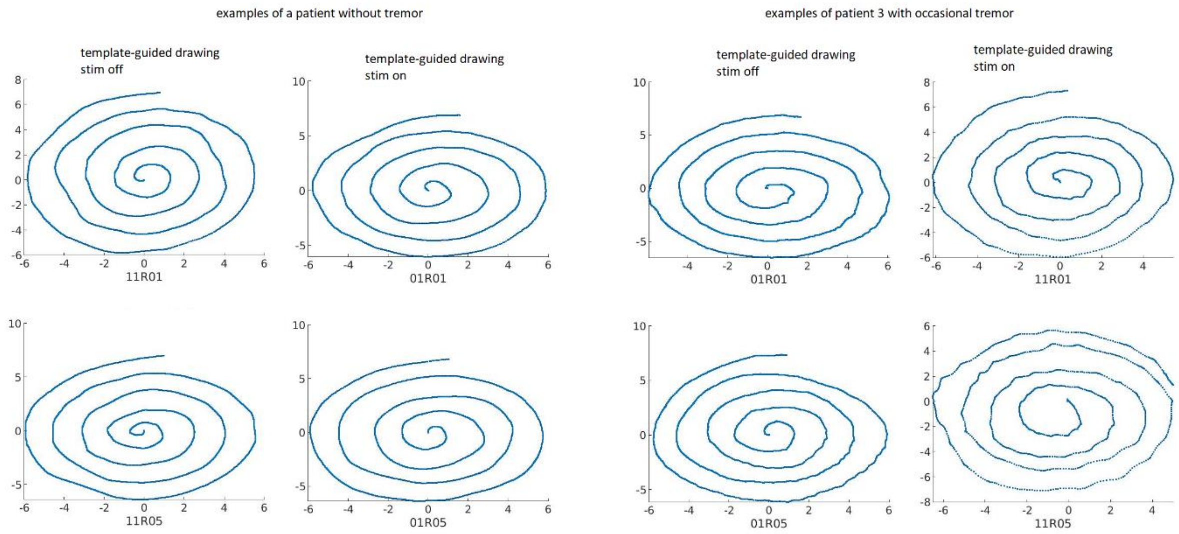

Supplementary Figure 7. Examples of spiral-drawings. One patient occasionally presented a slight tremor during drawing. Four template-guided drawings (each two trials with stimulation turned off and on) of a patient without action tremor are shown on the left, while four template-guided drawings (each two trials with stimulation turned off and on) of patient 3 who experienced occasional action tremor are shown on the right.

Supplementary Table 1. Demographics and disease related information.

Indication for surgery	Lead	Experimental order	Intensity contralateral (mA)	Intensity ipsilateral (mA)	Individual beta peak (Hz)	Template		No template	
						DBS on	DBS off	DBS on	DBS off
On-Off fluctuations/Tremor	Medtronic 3389™	2	5	5	23	+	+	+	+
On-Off fluctuations	Medtronic 3389™	2	1	1	18	+	+	+	+
On-Off fluctuations	Medtronic 3389™	2	1	1	18	+	+	na	na
On-Off fluctuations	Abbott 6170™	2	3.5	4	16	+	+	+	+
On-Off fluctuations	Abbott 6170™	2	2.5	no stim	19	+	+	+	+
On-Off fluctuations	Medtronic 3389™	1	3	no stim	18	+	+	+	+
On-Off fluctuations	Abbott 6170™	2	1.5	1.5	20	+	+	+	+
On-Off fluctuations	Abbott 6170™	1	2.4	2.4	20	+	+	+	+
On-Off fluctuations	Abbott 6170™	1	2.2	2.2	19	+	+	+	+
On-Off fluctuations	Abbott 6170™	2	2.6	2.6	20	+	+	na	+
On-Off fluctuations	Abbott 6170™	2	1.3	1.3	20	+	+	+	+
On-Off fluctuations	Abbott 6170™	2	1	1	22	+	+	+	+
Tremor	Abbott 6170™	1	2.7	2.7	20	+	+	+	+
On-Off fluctuations	Abbott 6170™	1	2.2	2.2	25	+	+	+	+
	Abbott 6170™	1	2.5	2.5	18	+	+	+	+
On-Off fluctuations	Abbott 6170™	1	2	2	18	+	+	+	+
	Abbott 6170™	2	3	3	17	+	+	+	+
On-Off fluctuations	Abbott 6170™	1	1.5	1.5	16	+	+	+	+
On-Off fluctuations	Abbott 6170™	1	1.7	1.7	14	+	+	+	+

ID	Sex	Handed-ness	Disease duration	H&Y pre	UPDRS pre (off meds)	UPDRS pre (on meds)	UPDRS pre delta	UPDRS (off DBS)	UPDRS (on DBS)	UPDRS delta	LEDD	Main symptom
1	m	r	5	2	8	5	3	36	28	8	880	Tremor
2	m	r	10	3	23	16	7	35	26	9	975	Bradykinesia
3	m	r	10	3	31	19	12	18	11	7	1348	Bradykinesia and Tremor
4	m	r	11	3.5	16	7	9	18	12	6	1270	Bradykinesia
5	f	r	13	2	18	15	3	11	11	0	1252	Bradykinesia and Tremor
6	m	r	7	4	35	29	6	17	17	0	675	Bradykinesia
7	m	r	6	3	33	14	19	22	17	5	1775	Bradykinesia and Freezing
8	f	r	5	2	25	17	8	24	20	4	1852	Bradykinesia
9	m	r	6	3	32	24	8	10	4	6	454	Tremor
10	m	r	12	4	59	50	9	32	29	3	1630	Bradykinesia
11	m	r	13	3.5	46	31	15	23	13	10	2128	Bradykinesia
12	m	r	16	5	44	20	24	23	13	10	950	Bradykinesia
13	m	r	3	3	34	32	2	27	21	6	505	Tremor
14	f	r	11	3	45	26	19	27	27	0	1275	Tremor
15	m	r	6	4	42	23	19	32	26	6	1536	Bradykinesia
16	m	l	11	3	24	9	15	36	20	16	440	Bradykinesia
17	m	r	15	3	62	54	8	39	27	12	653	Tremor
18	m	r	16	4	23	18	5	16	13	3	1683	Bradykinesia
19	f	r	22	3.5	49	37	12	32	27	5	1755	Bradykinesia

Data are disaggregated for sex (m = male, f = female). We did not obtain consent to publish information that identifies individuals and thus do not report exact age. Handedness is given as right (r) and left (l). Disease duration is given in years. Hoehn and Yahr (H&Y) stage was examined before surgery. Clinical

scores are given as total score of the MDS Unified Parkinson's disease rating scale (UPDRS) part III for levodopa off/on (before surgery) and as items 3-8 & 14-18 (limb scores) for DBS off/on. Medication is given in levodopa-equivalent daily dose (LEDD). Experimental order: on-off DBS (1), off-on DBS (2). Stimulation intensity for electrodes of the contralateral and ipsilateral STN (in relation to the drawing hand) are given in mA. Subject 3 and subject 10 were not able to complete all recordings due to fatigue (na: not available).

Supplementary Table 2. Exact cluster-statistics for the movement related desynchronization in in the beta band in both stimulation and drawing conditions.

drawing condition	stimulation condition	interval	cluster-number	cluster statistics	SD	CI range	*P*
free	off	early	1	-232.053	<0.001	0.001	0.001
free	off	early	2	-174.918	0.001	0.002	0.006
free	off	late	1	-321.926	0.001	0.002	0.004
free	off	late	2	-296.958	0.001	0.002	0.005
free	off	late	3	-140.574	0.002	0.004	0.018
free	off	late	4	-129.026	0.002	0.004	0.021
free	on	early	1	-264.435	0.001	0.003	0.010
free	on	early	2	-251.884	0.002	0.003	0.013
free	on	late	1	-184.277	0.002	0.005	0.027
template	off	early	1	-188.326	0.000	0.001	0.000
template	off	early	2	-80.760	0.003	0.006	0.043
template	off	late	1	-72.885	0.002	0.005	0.030
template	off	late	2	-69.128	0.003	0.005	0.035
template	on	early	1	-435.262	0.000	0.001	0.001
template	on	early	2	-268.146	0.001	0.003	0.009
template	on	early	3	-162.573	0.003	0.006	0.048
template	on	late	1	-210.178	0.002	0.004	0.021

Significant clusters of the one-sided cluster-based permutation test accounting for multiple comparisons (5000 permutations). Cluster-statistic: cluster-level test statistic (the sum of the T-values in this cluster); SD: standard deviation of the probability values; CI range: range of the confidence interval of the probability values. *P*: *P*-value

Supplementary Table 3. Associations between stimulation related reduction in burst amplitude and $UPDRS_{III}$ hand-motor scores assessed by spearman rank correlation (Bonferroni corrected for multiple comparisons).

	n	rho	*P*	*P* (corr)
Clinical associations				
ΔBurst amplitude (rest, free)	17	0.632	0.007	**0.026**
ΔBurst amplitude (draw, free)	16	0.634	0.008	**0.033**
ΔBurst amplitude (rest, template-guided)	19	0.625	0.004	**0.017**
ΔBurst amplitude (draw, template-guided)	18	0.614	0.007	**0.027**

Bold numbers indicate significant effects. n = number of samples; rho = spearman rho; *P* = *P*-value; *P* (corr) = Bonferroni-corrected *P*-value.

Supplementary Table 4. Effects of stimulation and drawing conditions on the drawing velocity, RMSE, and slope of the radius-angle transform (assessed by general linear models) on a single subject level in five participants showing only minor or no clinical improvement.

Tangential velocity (patient 5)	Estimate	SE	lower CI	upper CI	t(3/28)	*P*
stim	-0.110	0.021	-0.153	-0.067	-5.284	**<0.001**
template	-0.210	0.021	-0.253	-0.167	-10.088	**<0.001**
stim*template	0.058	0.029	-0.002	0.118	1.964	0.060

RMSE of radius-angle-transform (patient 5)	Estimate	SE	lower CI	upper CI	t(3/28)	*P*
stim	-0.342	0.062	-0.470	-0.214	-5.472	**<0.001**
template	-0.309	0.062	-0.437	-0.181	-4.950	**<0.001**
stim*template	0.341	0.088	0.160	0.522	3.862	**<0.001**

Slope of radius-angle-transform (patient 5)	Estimate	SE	lower CI	upper CI	t(3/28)	*P*
stim	-0.050	0.039	-0.129	0.030	-1.281	0.211
template	0.306	0.039	0.227	0.386	7.891	**<0.001**
stim*template	0.036	0.055	-0.076	0.148	0.656	0.517

Tangential velocity (patient 6)	Estimate	SE	lower CI	upper CI	t(3/36)	*P*
stim	0.026	0.031	-0.037	0.090	0.846	0.403
template	0.207	0.031	0.144	0.270	6.633	**<0.001**
stim*template	-0.048	0.044	-0.138	0.041	-1.098	0.279

RMSE of radius-angle-transform (patient 6)	Estimate	SE	lower CI	upper CI	t(3/12)	*P*
stim	0.465	0.284	-0.152	1.083	1.641	0.127
template	0.738	0.288	0.112	1.365	2.568	**0.025**
stim*template	-0.540	0.334	-1.267	0.187	-1.618	0.132

Slope of radius-angle-transform (patient 6)	Estimate	SE	lower CI	upper CI	t(3/36)	*P*
stim	-0.153	0.132	-0.420	0.114	-1.162	0.253
template	0.446	0.132	0.179	0.713	3.392	**0.002**
stim*template	0.284	0.186	-0.093	0.661	1.526	0.136

Tangential velocity (patient 10)	Estimate	SE	lower CI	upper CI	t(2/20)	*P*
stim	-0.064	0.041	-0.150	0.021	-1.573	0.131
template	-0.243	0.047	-0.342	-0.145	-5.143	**<0.001**
stim*template	na	na	na	na	na	na

RMSE of radius-angle-transform (patient 10)	Estimate	SE	lower CI	upper CI	T(2/10)	*P*
stim	-0.060	0.168	-0.433	0.314	-0.357	0.728
template	-0.456	0.153	-0.796	-0.115	-2.979	**0.014**
stim*template	na	na	na	na	na	na

Slope of radius-angle-transform (patient 10)	Estimate	SE	lower CI	upper CI	t(2/20)	*P*
stim	0.279	0.087	0.098	0.460	3.220	**0.004**
template	0.263	0.100	0.054	0.472	2.628	**0.016**
stim*template	na	na	na	na	na	na

Tangential velocity (patient 14)	Estimate	SE	lower CI	upper CI	t(3/36)	*P*
stim	0.293	0.022	0.248	0.337	13.341	**<0.001**
template	-0.159	0.022	-0.203	-0.114	-7.240	**<0.001**
stim*template	-0.158	0.031	-0.221	-0.095	-5.074	**<0.001**

RMSE of radius-angle-transform (patient 14)	Estimate	SE	lower Cl	upper Cl	t(3/34)	*P*
stim	0.539	0.103	0.330	0.749	5.229	**<0.001**
template	-0.019	0.106	-0.235	0.196	-0.182	0.856
stim*template	-0.611	0.150	-0.916	-0.307	-4.079	**<0.001**

Slope of radius-angle-transform (patient 14)	Estimate	SE	lower Cl	upper Cl	t(3/36)	*P*
stim	0.258	0.029	0.199	0.317	8.885	**<0.001**
template	0.183	0.029	0.124	0.242	6.299	**<0.001**
stim*template	-0.196	0.041	-0.280	-0.113	-4.782	**<0.001**

Tangential velocity (patient 18)	Estimate	SE	lower Cl	upper Cl	t(3/36)	*P*
stim	0.127	0.037	0.052	0.203	3.407	**0.002**
template	0.085	0.037	0.009	0.161	2.276	**0.029**
stim*template	-0.096	0.053	-0.203	0.011	-1.812	0.078

RMSE of radius-angle-transform (patient 18)	Estimate	SE	lower Cl	upper Cl	t(3/36)	*P*
stim	0.180	0.055	0.068	0.293	3.257	**0.002**
template	-0.026	0.055	-0.138	0.087	-0.465	0.645
stim*template	-0.142	0.078	-0.301	0.016	-1.820	0.077

Slope of radius-angle-transform (patient 18)	Estimate	SE	lower Cl	upper Cl	t(3/36)	*P*
stim	0.053	0.021	0.009	0.096	2.466	**0.019**
template	0.494	0.021	0.451	0.538	23.038	**<0.001**
stim*template	-0.056	0.030	-0.117	0.006	-1.838	0.074

Bold numbers indicate significant effects. SE = standard error; CI = Confidence interval; t = t-score (including degrees of freedom in brackets); *P* = *P*-value.

Supplementary Table 5. Detailed statistical results of the main analyses.

Main analyses of the manuscript

Statistical test							
Tangential velocity (log)[a]	Estimate	SE	lower CI	upper CI	df	t	*P*
stim	0.037	0.012	0.013	0.062	665.037	3.004	**0.003**
template	-0.168	0.012	-0.192	-0.144	665.268	-13.747	**<0.001**
stim*template	-0.026	0.017	-0.060	0.008	664.998	-1.497	0.135
RMSE of radius-angle-transform[a]	Estimate	SE	lower CI	upper CI	df	t	*P*
stim	0.054	0.028	0.000	0.108	626.253	1.958	0.051
template	-0.303	0.027	-0.356	-0.249	627.002	-11.055	**<0.001**
stim*template	-0.073	0.038	-0.148	0.003	625.625	-1.889	0.059
Slope of radius-angle-transform[a]	Estimate	SE	lower CI	upper CI	df	t	*P*
stim	0.051	0.016	0.019	0.082	665.328	3.121	**0.002**
template	0.291	0.016	0.260	0.322	666.578	18.376	**<0.001**
stim*template	-0.033	0.022	-0.077	0.011	665.106	-1.465	0.143
Tangential velocity (log) (controlling for RMSE)[a]	Estimate	SE	lower CI	upper CI	df	t	*P*
stim	0.023	0.011	0.001	0.044	624.080	2.062	**0.040**
template	-0.127	0.012	-0.150	-0.104	624.946	-10.658	**<0.001**
RMSE_glm	0.194	0.016	0.163	0.225	627.994	12.207	**<0.001**
stim*template	-0.003	0.015	-0.033	0.027	623.999	-0.209	0.834
Tangential velocity (log) (controlling for Slope)[a]	Estimate	SE	lower CI	upper CI	df	t	*P*
stim	0.025	0.012	0.002	0.048	664.131	2.104	**0.036**
template	-0.239	0.014	-0.267	-0.211	665.983	-16.790	**<0.001**
Slope_glm	0.244	0.028	0.189	0.300	669.288	8.619	**<0.001**
stim*template	-0.018	0.016	-0.050	0.014	664.017	-1.085	0.278
Beta Power[a]	Estimate	SE	lower CI	upper CI	df	t	*P*
stim	-0.071	0.010	-0.091	-0.051	1312	-7.018	**<0.001**
template	-0.021	0.010	-0.041	-0.002	1312	-2.163	**0.031**
interval	-0.049	0.010	-0.069	-0.030	1312	-4.979	**<0.001**
stim*template	0.013	0.014	-0.015	0.040	1312	0.904	0.366
stim*interval	0.031	0.014	0.003	0.059	1312	2.175	**0.030**
template*interval	0.011	0.014	-0.016	0.038	1312	0.773	0.440
stim*template*interval	-0.013	0.020	-0.051	0.026	1312	-0.637	0.524
Post hoc[b]	Estimated difference	SE	lower CI	upper CI	df	t-ratio	*P* (corrected)
rest_off - rest_on	0.065	0.007	0.046	0.083	1312	9.225	**<0.001**
rest_off - draw_off	0.044	0.007	0.025731	0.06239	1312	6.351	**<0.001**
rest_off - draw_on	0.084	0.007	0.065409	0.10236	1312	11.996	**<0.001**
rest_on - draw_off	-0.020	0.007	-0.03892	-0.00197	1312	-2.924	**0.021**
rest_on - draw_on	0.019	0.007	0.000827	0.03793	1312	2.760	**0.035**
draw_off - draw_on	0.040	0.007	0.021347	0.0583	1312	5.695	**<0.001**
Association between velocity and beta-ERD[a]	Estimate	SE	lower CI	upper CI	df	t	*P*
stim	0.036	0.014	0.009	0.063	643.256	2.583	**0.010**
template	-0.154	0.013	-0.180	-0.128	643.202	-11.527	**<0.001**
beta_diff	-0.122	0.087	-0.294	0.048	645.528	-1.402	0.161
stim*template	-0.023	0.019	-0.060	0.014	642.965	-1.212	0.226
stim*beta_ERD	-0.170	0.238	-0.635	0.294	643.996	-0.715	0.475

template*beta_ERD	0.302	0.117	0.073	0.531	643.256	2.576	**0.010**
stim*template*beta_ERD	0.149	0.323	-0.483	0.780	643.585	0.461	0.645

Post hoc (beta-ERD and velocity)[b]	Estimate	SE	lower CI	upper CI	df	t	*P*
free drawing	-0.426	0.384	-1.179	0.327	12.332	-1.109	0.289
template-guided drawing	0.016	0.066	-0.113	0.145	312.894	0.245	0.807

Gamma Power[a]	Estimate	SE	lower CI	upper CI	df	t	*P*
stim	0.000	0.003	-0.007	0.007	1312	-0.035	0.972
template	0.000	0.003	-0.007	0.006	1312	-0.126	0.900
interval	-0.001	0.003	-0.008	0.005	1312	-0.422	0.673
stim*template	0.000	0.005	-0.009	0.009	1312	0.011	0.991
stim*interval	0.007	0.005	-0.002	0.016	1312	1.478	0.140
template*interval	0.000	0.005	-0.009	0.009	1312	-0.022	0.982
stim*template*interval	-0.006	0.007	-0.019	0.007	1312	-0.923	0.356

Burst duration (log)[a]	Estimate	SE	lower CI	upper CI	df	t	*P*
stim	-0.013	0.014	-0.041	0.015	1264	-0.925	0.355
interval	-0.081	0.014	-0.109	-0.053	1265	-5.639	**<0.001**
template	0.006	0.014	-0.021	0.033	1264	0.459	0.646
stim*interval	0.042	0.020	0.002	0.082	1263	2.071	**0.039**
stim*template	-0.006	0.020	-0.044	0.033	1263	-0.283	0.777
interval*template	0.029	0.020	-0.010	0.068	1263	1.475	0.140
stim*interval*template	-0.026	0.028	-0.081	0.028	1263	-0.941	0.347

Post hoc[b]	Estimated difference	SE	lower CI	upper CI	df	z-ratio	*P* (corrected)
rest_off - draw_off	0.066	0.010	0.040	0.093	1269	6.581	**<0.001**
rest_off - rest_on	0.016	0.010	-0.010	0.042	1266	1.616	0.638
rest_off - draw_on	0.053	0.010	0.027	0.079	1268	5.368	**<0.001**
draw_off - rest_on	-0.050	0.010	-0.077	-0.024	1268	-4.978	**<0.001**
draw_off - draw_on	-0.013	0.010	-0.040	0.014	1267	-1.272	1.000
rest_on - draw_on	0.037	0.010	0.011	0.064	1266	3.759	**0.001**

Burst duration[a]	Estimate	SE	lower CI	upper CI	df	t	*P*
stim	-0.021	0.015	-0.050	0.008	1264.000	-1.419	0.156
interval	-0.060	0.015	-0.089	-0.030	1264	-4.001	**<0.001**
template	0.003	0.014	-0.025	0.032	1264	0.241	0.809
stim*interval	0.035	0.021	-0.006	0.076	1264	1.667	0.096
stim*template	0.008	0.020	-0.031	0.048	1264	0.407	0.684
interval*template	0.012	0.021	-0.028	0.053	1263	0.591	0.554
stim*interval*template	-0.025	0.029	-0.082	0.032	1264	-0.843	0.399

Burst amplitude (log)[a]	Estimate	SE	lower CI	upper CI	df	t	*P*
stim	-0.058	0.012	-0.080	-0.035	1265	-4.953	**<0.001**
interval	-0.051	0.012	-0.074	-0.028	1265	-4.305	**<0.001**
template	-0.020	0.011	-0.042	0.002	1265	-1.776	0.076
stim*interval	0.039	0.017	0.007	0.072	1265	2.362	**0.018**
stim*template	0.004	0.016	-0.028	0.035	1265	0.227	0.820
interval*template	0.014	0.016	-0.018	0.046	1265	0.834	0.405
stim*interval*template	-0.019	0.023	-0.064	0.026	1265	-0.820	0.413

Post hoc[b]	Estimated difference	SE	lower CI	upper CI	df	z-ratio	*P* (corrected)
rest_off - draw_off	0.044	0.008	0.022	0.066	1265	5.313	**<0.001**
rest_off - rest_on	0.056	0.008	0.034	0.077	1265	6.912	**<0.001**
rest_off - draw_on	0.070	0.008	0.048	0.091	1265	8.559	**<0.001**
draw_off - rest_on	0.012	0.008	-0.010	0.034	1265	1.434	0.911
draw_off - draw_on	0.026	0.008	0.004	0.048	1265	3.108	**0.012**

rest_on - draw_on	0.014	0.008	-0.008	0.036	1265	1.713	0.522

Burst amplitude[a]	Estimate	SE	lower CI	upper CI	df	t	*P*
stim	-0.339	0.047	-0.431	-0.247	1265.156	-7.219	**<0.001**
interval	-0.200	0.048	-0.293	-0.107	1265.196	-4.208	**<0.001**
template	-0.060	0.046	-0.150	0.029	1265.157	-1.319	0.188
stim*interval	0.178	0.067	0.046	0.310	1265.033	2.644	**0.008**
stim*template	0.044	0.065	-0.083	0.172	1265.014	0.680	0.497
interval*template	0.045	0.066	-0.085	0.174	1264.948	0.678	0.498
stim*interval*template	-0.067	0.093	-0.250	0.116	1264.969	-0.718	0.473

Number of bursts[a]	Estimate	SE	lower CI	upper CI	df	t	*P*
stim	0.238	0.389	-0.524	0.999	1265.372	0.612	0.541
interval	1.248	0.394	0.478	2.020	1265.569	3.165	**0.002**
template	-0.109	0.380	-0.852	0.635	1265.402	-0.286	0.775
stim*interval	0.868	0.558	-0.224	1.959	1264.776	1.556	0.120
stim*template	-0.064	0.540	-1.119	0.992	1264.684	-0.118	0.906
interval*template	5.910	0.549	4.836	6.983	1264.361	10.764	**<0.001**
stim*interval*template	-1.200	0.774	-2.715	0.315	1264.461	-1.550	0.121

Post hoc[b]	Estimated difference	SE	lower CI	upper CI	df	z-ratio	*P* (corrected)
rest_free - draw_free	-1.680	0.279	-2.420	-0.944	1266	-6.024	**<0.001**
rest_free - rest_template	0.140	0.271	-0.577	0.857	1267	0.518	1.000
rest_free - draw_template	-6.850	0.276	-7.581	-6.122	1268	-24.818	**<0.001**
draw_free - rest_template	1.820	0.277	1.091	2.554	1269	6.587	**<0.001**
draw_free - draw_template	-5.170	0.280	-5.908	-4.430	1267	-18.490	**<0.001**
rest_template - draw_template	-6.990	0.270	-7.705	-6.278	1267	-25.883	**<0.001**

Bursts per second[a]	Estimate	SE	lower CI	upper CI	df	t	*P*
stim	0.014	0.030	-0.045	0.074	1265.778	0.462	0.644
interval	-0.136	0.031	-0.196	-0.075	1266.043	-4.403	**<0.001**
template	-0.007	0.030	-0.065	0.051	1265.833	-0.248	0.804
stim*interval	0.047	0.044	-0.038	0.132	1264.985	1.082	0.279
stim*template	0.012	0.042	-0.071	0.095	1264.861	0.285	0.776
interval*template	0.028	0.043	-0.056	0.112	1264.429	0.642	0.521
stim*interval*template	-0.046	0.061	-0.164	0.073	1264.563	-0.755	0.450

Acceleration at burst onset[a]	Estimate	SE	lower CI	upper CI	df	t	*P*
stim	0.013	0.004	0.005	0.020	10880	3.204	**0.001**
template	0.003	0.004	-0.004	0.010	10870	0.816	0.415
interval	0.014	0.004	0.006	0.022	10850	3.388	**<0.001**
stim*template	-0.013	0.005	-0.022	-0.004	10870	-2.713	**0.007**
stim*interval	-0.007	0.006	-0.018	0.004	10850	-1.315	0.189
template*interval	-0.012	0.005	-0.021	-0.002	10850	-2.348	**0.019**
stim*template*interval	0.012	0.007	-0.001	0.025	10850	1.781	0.075

Acceleration at burst onset (controlling for burstrate, amplitude, duration during rest)[a]	Estimate	SE	lower CI	upper CI	df	t	*P*
stim	0.013	0.004	0.005	0.020	10860	3.228	**0.001**
template	0.003	0.004	-0.004	0.010	10830	0.885	0.376
interval	0.014	0.004	0.006	0.022	10840	3.388	**0.001**
Burstrate_rest	0.004	0.003	-0.001	0.010	10790	1.527	0.127
duration_rest_Log	-0.006	0.007	-0.019	0.007	2678	-0.939	0.348
amplitude_rest_Log	0.008	0.008	-0.006	0.023	117.300	1.102	0.273
stim*template	-0.013	0.005	-0.023	-0.004	10860	-2.767	**0.006**
stim*interval	-0.007	0.006	-0.018	0.004	10840	-1.315	0.189
template*interval	-0.012	0.005	-0.021	-0.002	10840	-2.348	**0.019**

stim*template*interval	0.012	0.007	-0.001	0.025	10840	1.810	0.070

Acceleration at burst onset (controlling for amplitude during rest)[a]	Estimate	SE	lower CI	upper CI	df	t	*P*
stim	0.013	0.004	0.005	0.020	10860	3.235	**0.001**
template	0.003	0.004	-0.004	0.010	10840	0.888	0.374
interval	0.014	0.004	0.006	0.022	10840	3.388	**0.001**
amplitude_rest_Log	0.006	0.007	-0.008	0.020	160	0.853	0.395
stim*template	-0.013	0.005	-0.023	-0.004	10860	-2.771	**0.006**
stim*interval	-0.007	0.006	-0.018	0.004	10840	-1.315	0.189
template*interval	-0.012	0.005	-0.021	-0.002	10840	-2.348	**0.019**
stim*template*interval	0.012	0.007	-0.001	0.025	10840	1.810	0.070

Acceleration at burst onset (controlling for duration during rest)[a]	Estimate	SE	lower CI	upper CI	df	t	*P*
stim	0.013	0.004	0.005	0.020	10860	3.189	**0.001**
template	0.003	0.004	-0.004	0.010	10860	0.800	0.424
interval	0.014	0.004	0.006	0.022	10840	3.388	**0.001**
duration_rest_Log	-0.004	0.006	-0.016	0.008	10390	-0.701	0.483
stim*template	-0.013	0.005	-0.022	-0.004	10860	-2.707	**0.007**
stim*interval	-0.007	0.006	-0.018	0.004	10840	-1.315	0.189
template*interval	-0.012	0.005	-0.021	-0.002	10840	-2.348	**0.019**
stim*template*interval	0.012	0.007	-0.001	0.025	10840	1.810	0.070

Acceleration at burst onset (controlling for burstrate during rest)[a]	Estimate	SE	lower CI	upper CI	df	t	*P*
stim	0.013	0.004	0.005	0.020	10860	3.206	**0.001**
template	0.003	0.004	-0.004	0.010	10860	0.803	0.422
interval	0.014	0.004	0.006	0.022	10840	3.388	**0.001**
nBursts_rest	0.005	0.003	-0.001	0.010	10680	1.640	0.101
stim*template	-0.013	0.005	-0.023	-0.004	10860	-2.732	**0.006**
stim*interval	-0.007	0.006	-0.018	0.004	10840	-1.315	0.189
template*interval	-0.012	0.005	-0.021	-0.002	10840	-2.348	**0.019**
stim*template*interval	0.012	0.007	-0.001	0.025	10840	1.810	0.070

Post hoc (interval*template)[b]	Estimated difference	SE	lower Cl	upper Cl	df	z-ratio	*P* (corrected)
free_post - template_post	0.004	0.002	-0.003	0.010	10868	1.503	0.796
free_post - free_pre	-0.010	0.003	-0.017	-0.003	10859	-3.660	**0.002**
free_post - template_pre	-0.001	0.002	-0.007	0.005	10868	-0.402	1.000
template_post - free_pre	-0.014	0.002	-0.020	-0.007	10868	-5.689	**<0.001**
template_post - template_pre	-0.005	0.002	-0.010	0.000	10859	-2.383	0.103
free_pre - template_pre	0.009	0.002	0.003	0.016	10868	3.784	**0.001**

Post hoc (stim*template)[b]	Estimated difference	SE	lower Cl	upper Cl	df	z-ratio	*P* (corrected)
free_off - template_off	0.003	0.003	-0.004	0.010	10843	1.157	1.000
free_off - free_on	-0.009	0.003	-0.016	-0.002	10863	-3.205	**0.008**
free_off - template_on	0.001	0.002	-0.006	0.007	10847	0.369	1.000
template_off - free_on	-0.012	0.002	-0.018	-0.006	10752	-4.970	**<0.001**
template_off - template_on	-0.002	0.002	-0.007	0.003	10868	-1.013	1.000
free_on - template_on	0.010	0.002	0.004	0.016	10862	4.217	**<0.001**

Acceleration at burst onset (linear bursts)[a]	Estimate	SE	lower CI	upper CI	df	t	*P*
stim	0.001	0.002	-0.002	0.005	53450	0.746	0.456
template	-0.006	0.002	-0.010	-0.003	53410	-4.055	**<0.001**
interval	0.003	0.002	-0.001	0.006	53430	1.466	0.143
stim*template	-0.001	0.002	-0.005	0.004	53440	-0.234	0.815

stim*interval	0.000	0.003	-0.005	0.005	53430	0.109	0.913
template*interval	-0.002	0.002	-0.006	0.003	53430	-0.685	0.493
stim*template*interval	0.000	0.003	-0.006	0.006	53430	0.071	0.943

Acceleration at burst onset (random bursts)[a]	Estimate	SE	lower CI	upper CI	df	t	*P*
stim	0.005	0.004	-0.002	0.013	10990	1.333	0.183
template	0.000	0.003	-0.007	0.006	10990	-0.108	0.914
interval	0.005	0.004	-0.003	0.012	10970	1.137	0.256
stim*template	-0.004	0.005	-0.013	0.006	10980	-0.772	0.440
stim*interval	-0.001	0.005	-0.012	0.010	10970	-0.168	0.867
template*interval	-0.004	0.005	-0.013	0.006	10970	-0.777	0.437
stim*template*interval	-0.001	0.007	-0.014	0.012	10970	-0.163	0.870

Residual at burst onset[a]	Estimate	SE	lower CI	upper CI	df	t	*P*
stim	0.001	0.001	-0.001	0.003	8426	0.980	0.327
template	0.000	0.001	-0.002	0.002	6555	0.103	0.918
interval	0.000	0.001	-0.002	0.002	8946	0.000	1.000
stim*template	-0.001	0.001	-0.003	0.001	8814	-0.902	0.367
stim*interval	0.000	0.001	-0.003	0.003	8946	0.000	1.000
template*interval	0.000	0.001	-0.002	0.002	8946	0.000	1.000
stim*template*interval	0.000	0.002	-0.003	0.003	8946	0.000	1.000

Residual at burst onset (controlling for burstrate, amplitude, duration during rest)[a]	Estimate	SE	lower CI	upper CI	df	t	*P*
stim	0.001	0.001	-0.001	0.003	8517	0.984	0.325
template	0.000	0.001	-0.002	0.002	6683	0.165	0.869
interval	0.000	0.001	-0.002	0.002	8937	0.000	1.000
Burstrate_rest	-0.001	0.001	-0.002	0.001	4428	-0.878	0.380
duration_rest_Log	-0.001	0.001	-0.004	0.002	833	-0.800	0.424
amplitude_rest_Log	0.000	0.001	-0.001	0.002	10	0.485	0.638
stim*template	-0.001	0.001	-0.003	0.001	8774	-0.893	0.372
stim*interval	0.000	0.001	-0.003	0.003	8937	0.000	1.000
template*interval	0.000	0.001	-0.002	0.002	8937	0.000	1.000
stim*template*interval	0.000	0.002	-0.003	0.003	8937	0.000	1.000

Residual at burst onset (controlling for burstrate during rest)[a]	Estimate	SE	lower CI	upper CI	df	t	*P*
stim	0.001	0.001	-0.001	0.003	8436	0.972	0.331
template	0.000	0.001	-0.002	0.002	6493	0.112	0.911
interval	0.000	0.001	-0.002	0.002	8934	0.000	1.000
Burstrate_rest	0.000	0.001	-0.002	0.001	2017	-0.748	0.454
stim*template	-0.001	0.001	-0.003	0.001	8801	-0.894	0.371
stim*interval	0.000	0.001	-0.003	0.003	8934	0.000	1.000
template*interval	0.000	0.001	-0.002	0.002	8934	0.000	1.000
stim*template*interval	0.000	0.002	-0.003	0.003	8934	0.000	1.000

Residual at burst onset (controlling for duration during rest)[a]	Estimate	SE	lower CI	upper CI	df	t	*P*
stim	0.001	0.001	-0.001	0.003	8389	0.970	0.332
template	0.000	0.001	-0.002	0.002	6495	0.092	0.927
interval	0.000	0.001	-0.002	0.002	8936	0.000	1.000
duration_rest_Log	-0.001	0.001	-0.004	0.002	1378	-0.656	0.512
stim*template	-0.001	0.001	-0.003	0.001	8765	-0.877	0.381
stim*interval	0.000	0.001	-0.003	0.003	8936	0.000	1.000
template*interval	0.000	0.001	-0.002	0.002	8936	0.000	1.000
stim*template*interval	0.000	0.002	-0.003	0.003	8936	0.000	1.000

Residual at burst onset (controlling for amplitude during rest)[a]	Estimate	SE	lower CI	upper CI	df	t	*P*
stim	0.001	0.001	-0.001	0.003	8493	0.997	0.319
template	0.000	0.001	-0.002	0.002	6627	0.153	0.878
interval	0.000	0.001	-0.002	0.002	8938	0.000	1.000
amplitude_rest_Log	0.000	0.001	-0.001	0.002	11	0.316	0.758
stim*template	-0.001	0.001	-0.003	0.001	8767	-0.916	0.360
stim*interval	0.000	0.001	-0.003	0.003	8938	0.000	1.000
template*interval	0.000	0.001	-0.002	0.002	8938	0.000	1.000
stim*template*interval	0.000	0.002	-0.003	0.003	8938	0.000	1.000

Associatons Δaccerelation and burst amplitude[a]	Estimate	SE	lower CI	upper CI	df	t	*P*
stim	-0.004	0.005	-0.013	0.005	1432	-0.974	0.330
template	0.008	0.005	-0.001	0.017	4354	1.752	0.080
amplitudeLog	0.011	0.016	-0.022	0.045	213	0.688	0.492
stim*amplitudeLog	-0.058	0.022	-0.102	-0.015	4749	-2.628	**0.009**
template*amplitudeLog	-0.013	0.019	-0.049	0.024	5157	-0.674	0.500
stim*template*amplitudeLog	0.053	0.025	0.004	0.103	5239	2.103	**0.036**

Associatons Δaccerelation and burst amplitude (incl. covariates)[a]	Estimate	SE	lower CI	upper CI	df	t	*P*
stim	-0.005	0.005	-0.014	0.004	2118	-1.085	0.278
template	0.008	0.005	-0.002	0.017	4633	1.660	0.097
amplitudeLog	0.027	0.025	-0.022	0.076	1072	1.065	0.287
Burstrate_rest	-0.010	0.006	-0.022	0.003	4786	-1.566	0.117
duration_rest_Log	0.031	0.015	0.000	0.060	2711	2.004	**0.045**
amplitude_rest_Log	-0.018	0.021	-0.058	0.024	825.6	-0.848	0.397
stim*amplitudeLog	-0.060	0.022	-0.103	-0.016	5009	-2.667	**0.008**
template*amplitudeLog	-0.013	0.019	-0.050	0.023	5425	-0.724	0.469
stim*template*amplitudeLog	0.052	0.025	0.002	0.102	5426	2.047	**0.041**

Post hoc: Δaccerelation and burst amplitude[a]	Estimate	SE	lower CI	upper CI	df	t	*P*
free_off	0.014	0.020			825	0.709	0.479
free_on	-0.043	0.032	-0.107	0.024	9.999	-1.331	0.213
template_off	0.004	0.009	-0.015	0.022	1794	0.401	0.688
template_on	0.000	0.018			7.899	0.016	0.988

Post hoc: Δaccerelation and burst amplitude (incl. covariates)[a]	Estimate	SE	lower CI	upper CI	df	t	*P*
amplitudeLog	0.018	0.020			823.965	0.923	0.356
duration_rest_Log	0.075	0.043			824	1.742	0.082
amplitudeLog	-0.041	0.032			10.724	-1.282	0.227
duration_rest_Log	0.035	0.053			702.486	0.656	0.512
amplitudeLog	0.004	0.009	-0.014	0.023	0.060	0.460	0.921
duration_rest_Log	0.012	0.018	-0.023	0.047	4.062	0.684	0.531
amplitudeLog	-0.002	0.017	-0.035	0.033	10.330	-0.138	0.893
duration_rest_Log	0.016	0.019	-0.021	0.054	1121	0.815	0.415

Associatons Δaccerelation and burst duration[a]	Estimate	SE	lower CI	upper CI	df	t	*P*
stim	-0.003	0.012	-0.026	0.020	5402	-0.246	0.806
template	0.005	0.013	-0.020	0.030	5353	0.401	0.688
durationLog	0.012	0.019	-0.026	0.050	1308	0.634	0.526
stim*durationLog	-0.016	0.019	-0.053	0.022	5428	-0.822	0.411
template*durationLog	-0.011	0.020	-0.050	0.028	5209	-0.556	0.578
stim*template*durationLog	0.019	0.012	-0.004	0.042	5394	1.645	0.100

Associatons Δaccerelation and burst duration (incl. covariates)[a]	Estimate	SE	lower CI	upper CI	df	t	*P*
stim	-0.004	0.012	-0.027	0.019	5318	-0.306	0.760
template	0.004	0.013	-0.021	0.029	5234	0.338	0.736
durationLog	0.013	0.019	-0.025	0.051	1308	0.696	0.486
burstrate_rest	-0.009	0.006	-0.022	0.003	4671	-1.465	0.143
duration_rest_Log	0.025	0.014	-0.003	0.053	1246	1.774	0.076
amplitude_rest_Log	-0.002	0.010	-0.021	0.018	19	-0.189	0.852
stim*durationLog	-0.017	0.019	-0.054	0.021	5419	-0.883	0.378
template*durationLog	-0.013	0.020	-0.052	0.027	5295	-0.635	0.525
stim*template*durationLog	0.020	0.012	-0.003	0.043	5368	1.711	0.087

Bold numbers indicate significant effects. SE = standard error; CI = Confidence interval; df = degrees of freedom; t = t-score; *P* = *P*-value; *P* (corr) = Bonferroni-corrected *P*-value.

[a] two-sided linear mixed-effects model

[b] two-sided pairwise estimated marginal means post hoc test (Bonferroni corrected for multiple comparisons)

Supplementary Table 6. Detailed statistical results of the analyses excluding inaccurate trials and tremor-dominant patients.

Main analyses excluding inaccurate trials

Statistical test (excluded inaccurate trials)							
Tangential velocity (log)[a]	Estimate	SE	lower CI	upper CI	df	t	*P*
stim	0.051	0.013	0.024	0.077	530.418	3.789	**<0.001**
template	-0.160	0.013	-0.185	-0.135	529.877	-12.543	**<0.001**
stim*template	-0.042	0.018	-0.077	-0.008	529.777	-2.381	**0.018**
RMSE of radius-angle-transform[a]	Estimate	SE	lower CI	upper CI	df	t	*P*
stim	0.067	0.027	0.015	0.120	520.981	2.498	**0.013**
template	-0.317	0.026	-0.368	-0.266	519.960	-12.255	**<0.001**
stim*template	-0.089	0.036	-0.160	-0.018	519.230	-2.452	**0.015**
Slope of radius-angle-transform[a]	Estimate	SE	lower CI	upper CI	df	t	*P*
stim	0.060	0.015	0.030	0.089	537.513	3.998	**<0.001**
template	0.283	0.014	0.255	0.311	538.404	19.862	**<0.001**
stim*template	-0.053	0.020	-0.092	-0.014	533.914	-2.650	**0.008**
Tangential velocity (log) (controlling for RMSE)[a]	Estimate	SE	lower CI	upper CI	df	t	*P*
stim1	0.036	0.012	0.012	0.060	515.942	2.969	**0.003**
template1	-0.089	0.013	-0.115	-0.063	517.093	-6.738	**<0.001**
RMSE_glm	0.223	0.020	0.185	0.262	519.281	11.353	**<0.001**
stim1*template1	-0.023	0.016	-0.055	0.009	515.516	-1.415	0.158
Tangential velocity (log) (controlling for Slope)[a]	Estimate	SE	lower CI	upper CI	df	t	*P*
stim1	0.025	0.012	0.001	0.049	529.563	2.078	**0.038**
template1	-0.273	0.015	-0.303	-0.244	529.381	-18.209	**<0.001**
Slope_glm	0.401	0.034	0.333	0.468	530.278	11.622	**<0.001**
stim1*template1	-0.020	0.016	-0.051	0.012	528.832	-1.232	0.219
Burst duration (log)[a]	Estimate	SE	lower CI	upper CI	df	t	*P*
stim	-0.011	0.016	-0.042	0.020	1031	-0.684	0.494
interval	-0.089	0.016	-0.121	-0.057	1025	-5.468	**<0.001**
template	-0.002	0.015	-0.032	0.028	1033	-0.151	0.880
stim*interval	0.034	0.023	-0.010	0.079	1025	1.515	0.130
stim*template	-0.014	0.021	-0.055	0.028	1028	-0.646	0.519
interval*template	0.037	0.022	-0.005	0.080	1027	1.717	0.086
stim*interval*template	-0.015	0.030	-0.074	0.044	1025	-0.496	0.620
Burst amplitude (log)[a]	Estimate	SE	lower CI	upper CI	df	t	*P*
stim	-0.095	0.013	-0.121	-0.070	1029	-7.319	**<0.001**
interval	-0.054	0.013	-0.080	-0.028	1029	-4.090	**<0.001**
template	-0.041	0.013	-0.066	-0.017	1029	-3.292	**<0.001**
stim*interval	0.036	0.018	0.000	0.072	1029	1.943	0.052
stim*template	0.027	0.017	-0.007	0.061	1029	1.531	0.126
interval*template	0.018	0.018	-0.017	0.053	1029	1.017	0.309
stim*interval*template	-0.015	0.025	-0.063	0.034	1029	-0.597	0.551
Post hoc[b]	Estimated difference	SE	lower CI	upper CI	df	z-ratio	*P* (corrected)
rest_off - draw_off	0.045	0.009	0.022	0.069	1029	5.099	**<0.001**
rest_off - rest_on	0.082	0.009	0.059	0.105	1029	9.435	**<0.001**
rest_off - draw_on	0.099	0.009	0.076	0.122	1029	11.281	**<0.001**
draw_off - rest_on	0.037	0.009	0.013	0.060	1029	4.123	**<0.001**

draw_off - draw_on	0.054	0.009	0.030	0.077	1029	5.997	**<0.001**
rest_on - draw_on	0.017	0.009	-0.006	0.040	1029	1.952	0.307
Number of bursts[a]	Estimate	SE	lower CI	upper CI	df	t	*P*
stim	0.202	0.454	-0.686	1.089	1033.005	0.446	0.656
interval	1.663	0.464	0.757	2.570	1029.004	3.587	**<0.001**
template	-0.325	0.436	-1.176	0.527	1034.258	-0.746	0.456
stim*interval	0.283	0.643	-0.975	1.540	1028.925	0.440	0.660
stim*template	-0.040	0.605	-1.223	1.143	1031.112	-0.066	0.947
interval*template	5.594	0.619	4.385	6.805	1030.023	9.037	**<0.001**
stim*interval*template	-1.000	0.863	-2.688	0.688	1028.895	-1.158	0.247
Post hoc[b]	Estimated difference	SE	lower CI	upper CI	df	z-ratio	*P* (corrected)
rest_free - draw_free	-1.805	0.322	-2.655	-0.954	1029	-5.611	**<0.001**
rest_free - rest_template	0.345	0.310	-0.473	1.163	1039	1.115	1.000
rest_free - draw_template	-6.554	0.312	-7.379	-5.728	1036	-20.986	**<0.001**
draw_free - rest_template	2.149	0.313	1.323	2.976	1039	6.874	**<0.001**
draw_free - draw_template	-4.749	0.315	-5.582	-3.916	1035	-15.073	**<0.001**
rest_template - draw_template	-6.899	0.291	-7.667	-6.130	1033	-23.736	**<0.001**
Bursts per second[a]	Estimate	SE	lower CI	upper CI	df	t	*P*
stim	0.025	0.035	-0.043	0.093	1034	0.718	0.473
interval	-0.137	0.035	-0.206	-0.067	1029	-3.858	**<0.001**
template	0.008	0.033	-0.057	0.073	1036	0.241	0.810
stim*interval	0.030	0.049	-0.066	0.126	1029	0.605	0.545
stim*template	-0.013	0.046	-0.103	0.078	1031	-0.275	0.783
interval*template	0.045	0.047	-0.048	0.137	1030	0.946	0.344
stim*interval*template	-0.034	0.066	-0.163	0.095	1029	-0.514	0.607
Acceleration at burst onset[a]	Estimate	SE	lower CI	upper CI	df	t	*P*
stim	0.013	0.004	0.005	0.022	8989	3.120	**0.002**
template	0.001	0.004	-0.006	0.009	8998	0.368	0.713
interval	0.011	0.004	0.002	0.019	9021	2.430	**0.015**
stim*template	-0.015	0.005	-0.025	-0.005	9034	-2.983	**0.003**
stim*interval	-0.008	0.006	-0.020	0.004	9021	-1.294	0.196
template*interval	-0.010	0.005	-0.020	0.000	9021	-1.906	0.057
stim*template*interval	0.014	0.007	0.000	0.028	9021	1.899	0.058
controlling for burstrate, amplitude, duration during rest[a]	Estimate	SE	lower CI	upper CI	df	t	*P*
stim	0.014	0.004	0.006	0.022	8719	3.234	**0.001**
template	0.002	0.004	-0.006	0.009	8867	0.475	0.635
interval	0.011	0.004	0.002	0.019	9007	2.430	**0.015**
Burstrate_rest	0.004	0.003	-0.002	0.009	8822	1.226	0.220
duration_rest_Log	-0.006	0.007	-0.020	0.008	1952	-0.864	0.388
amplitude_rest_Log	0.012	0.008	-0.004	0.028	66	1.410	0.163
stim*template	-0.016	0.005	-0.026	-0.006	8973	-3.065	**0.002**
stim*interval	-0.008	0.006	-0.020	0.004	9007	-1.294	0.196
template*interval	-0.010	0.005	-0.020	0.000	9007	-1.905	0.057
stim*template*interval	0.014	0.007	0.000	0.028	9007	1.932	0.053
controlling for amplitude during rest[a]	Estimate	SE	lower CI	upper CI	df	t	*P*
stim	0.014	0.004	0.005	0.022	8757	3.205	**0.001**
template	0.002	0.004	-0.006	0.009	8891	0.477	0.633
interval	0.011	0.004	0.002	0.019	9009	2.430	**0.015**
amplitude_rest_Log	0.009	0.008	-0.006	0.024	88	1.153	0.252
stim*template	-0.016	0.005	-0.026	-0.006	8987	-3.050	**0.002**
stim*interval	-0.008	0.006	-0.020	0.004	9009	-1.294	0.196

template*interval	-0.010	0.005	-0.020	0.000	9009	-1.905	0.057
stim*template*interval	0.014	0.007	0.000	0.028	9009	1.932	0.053
controlling for duration during rest[a]	Estimate	SE	lower CI	upper CI	df	t	*P*
stim	0.013	0.004	0.005	0.022	8979	3.114	**0.002**
template	0.001	0.004	-0.006	0.009	8986	0.359	0.720
interval	0.011	0.004	0.002	0.019	9010	2.430	**0.015**
duration_rest_Log	-0.004	0.007	-0.017	0.009	8096	-0.554	0.580
stim*template	-0.015	0.005	-0.026	-0.005	9023	-2.999	**0.003**
stim*interval	-0.008	0.006	-0.020	0.004	9010	-1.294	0.196
template*interval	-0.010	0.005	-0.020	0.000	9010	-1.905	0.057
stim*template*interval	0.014	0.007	0.000	0.028	9010	1.932	0.053
controlling for burstrate during rest[a]	Estimate	SE	lower CI	upper CI	df	t	*P*
stim	0.013	0.004	0.005	0.022	8978	3.135	**0.002**
template	0.001	0.004	-0.006	0.009	8984	0.348	0.728
interval	0.011	0.004	0.002	0.019	9009	2.430	**0.015**
Burstrate_rest	0.004	0.003	-0.002	0.010	8758	1.279	0.201
stim*template	-0.015	0.005	-0.026	-0.005	9023	-2.999	**0.003**
stim*interval	-0.008	0.006	-0.020	0.004	9009	-1.294	0.196
template*interval	-0.010	0.005	-0.020	0.000	9009	-1.905	0.057
stim*template*interval	0.014	0.007	0.000	0.028	9009	1.932	0.053
Post hoc (interval*template)[b]	Estimated difference	SE	lower CI	upper CI	df	z-ratio	*P* (corrected)
free_post - template_post	0.006	0.003	-0.001	0.013	8918	2.404	0.097
free_post - free_pre	-0.007	0.003	-0.015	0.001	9027	-2.267	0.140
free_post - template_pre	0.003	0.003	-0.004	0.009	8918	0.980	1.000
template_post - free_pre	-0.013	0.003	-0.020	-0.006	8918	-5.013	**<0.001**
template_post - template_pre	-0.004	0.002	-0.009	0.002	9027	-1.869	0.370
free_pre - template_pre	0.009	0.003	0.002	0.016	8918	3.590	**0.002**
Post hoc (stim*template)[b]	Estimated difference	SE	lower CI	upper CI	df	z-ratio	*P* (corrected)
free_off - template_off	0.004	0.003	-0.004	0.010	8828	1.316	1.000
free_off - free_on	-0.010	0.003	-0.018	-0.002	8786	-3.098	**0.012**
free_off - template_on	0.003	0.003	-0.005	0.009	8960	0.948	1.000
template_off - free_on	-0.013	0.003	-0.020	-0.006	7889	-5.009	**<0.001**
template_off - template_on	-0.001	0.002	-0.007	0.004	8942	-0.500	1.000
free_on - template_on	0.012	0.003	0.005	0.019	8641	4.751	**<0.001**
Residual at burst onset[a]	Estimate	SE	lower CI	upper CI	Df	t	*P*
stim	0.001	0.001	-0.001	0.002	7544	0.562	0.574
template	-0.001	0.001	-0.002	0.001	7544	-0.655	0.512
interval	0.000	0.001	-0.002	0.002	7544	0.000	1.000
stim*template	-0.001	0.001	-0.003	0.002	7544	-0.518	0.604
stim*interval	0.000	0.001	-0.003	0.003	7544	0.000	1.000
template*interval	0.000	0.001	-0.002	0.002	7544	0.000	1.000
stim*template*interval	0.000	0.002	-0.003	0.003	7544	0.000	1.000
Associatons Δaccerelation and burst amplitude[a]	Estimate	SE	lower CI	upper CI	Df	t	*P*
stim	-0.004	0.005	-0.013	0.006	2405	-0.739	0.460
template	0.007	0.005	-0.003	0.017	2523	1.442	0.149
amplitudeLog	-0.007	0.017	-0.041	0.026	192	-0.416	0.678
stim*amplitudeLog	-0.037	0.023	-0.082	0.008	4036	-1.591	0.112
template*amplitudeLog	0.004	0.019	-0.034	0.042	3522	0.224	0.822
stim*template*amplitudeLog	0.038	0.026	-0.013	0.089	3956	1.455	0.146

Associatons Δaccerelation and burst amplitude (incl. covariates)[a]	Estimate	SE	lower CI	upper CI	Df	t	*P*
stim	-0.005	0.005	-0.014	0.005	2867	-0.935	0.350
template	0.007	0.005	-0.003	0.017	2830	1.314	0.189
amplitudeLog	0.021	0.027	-0.031	0.074	1096	0.781	0.435
Burstrate_rest	-0.006	0.007	-0.020	0.007	3666	-0.963	0.336
duration_rest_Log	0.030	0.016	-0.002	0.062	2128	1.854	0.064
amplitude_rest_Log	-0.029	0.022	-0.072	0.014	639	-1.314	0.189
stim*amplitudeLog	-0.039	0.023	-0.084	0.006	4207	-1.688	0.092
template*amplitudeLog	0.003	0.019	-0.035	0.041	4438	0.152	0.879
stim*template*amplitudeLog	0.036	0.026	-0.015	0.087	4437	1.380	0.168

Main analyses excluding tremor-dominant patients

Statistical test (excluded 7 tremor-dominant patients)

Tangential velocity (log)[a]	Estimate	SE	df	lower CI	upper CI	t	*P*
stim	0.048	0.016	413.124	0.016	0.080	2.927	**0.004**
template	-0.139	0.016	412.972	-0.171	-0.108	-8.648	**<0.001**
stim*template	-0.053	0.023	413.010	-0.098	-0.009	-2.339	**0.020**

RMSE of radius-angle-transform[a]	Estimate	SE	lower CI	upper CI	df	t	*P*
stim	0.078	0.039	0.002	0.154	395.309	2.017	**0.044**
template	-0.321	0.038	-0.397	-0.246	394.696	-8.358	**<0.001**
stim*template	-0.106	0.054	-0.212	0.000	394.655	-1.964	0.050

Slope of radius-angle-transform[a]	Estimate	SE	lower CI	upper CI	df	t	*P*
stim	0.049	0.023	0.005	0.093	432.219	2.160	**0.031**
template	0.304	0.022	0.261	0.347	432.192	13.779	**<0.001**
stim*template	-0.028	0.031	-0.089	0.033	432.014	-0.893	0.372

Tangential velocity (log) (controlling for RMSE)[a]	Estimate	SE	lower CI	upper CI	df	t	*P*
stim	0.028	0.015	0.000	0.057	393.100	1.927	0.055
template	-0.104	0.016	-0.135	-0.074	393.331	-6.650	**<0.001**
RMSE_glm	0.190	0.019	0.153	0.227	394.907	10.061	**<0.001**
stim*template	-0.022	0.020	-0.062	0.018	393.035	-1.064	0.288

Tangential velocity (log) (controlling for Slope)[a]	Estimate	SE	lower CI	upper CI	df	t	*P*
stim	0.037	0.016	0.006	0.068	431.074	2.365	**0.019**
template	-0.206	0.018	-0.242	-0.170	431.911	-11.198	**<0.001**
Slope_glm	0.221	0.033	0.155	0.286	433.890	6.603	**<0.001**
stim*template	-0.047	0.022	-0.090	-0.005	431.007	-2.164	**0.031**

Burst duration (log)[a]	Estimate	SE	lower CI	upper CI	df	t	*P*
stim	-0.022	0.018	-0.056	0.012	795.063	-1.247	0.213
interval	-0.078	0.018	-0.113	-0.043	795.267	-4.336	**<0.001**
template	0.004	0.017	-0.029	0.038	794.144	0.242	0.809
stim*interval	0.042	0.025	-0.007	0.092	794.526	1.658	0.098
stim*template	0.012	0.025	-0.036	0.060	794.417	0.481	0.631
interval*template	0.039	0.025	-0.010	0.088	794.131	1.540	0.124
stim*interval*template	-0.058	0.035	-0.127	0.011	794.218	-1.637	0.102

Burst amplitude (log)[a]	Estimate	SE	lower CI	upper CI	df	t	*P*
stim	-0.044	0.016	-0.076	-0.012	795.067	-2.670	**0.008**
interval	-0.056	0.017	-0.088	-0.023	795.084	-3.318	**<0.001**
template	-0.022	0.016	-0.053	0.010	794.995	-1.362	0.174
stim*interval	0.050	0.024	0.004	0.097	795.025	2.127	**0.034**

stim*template	0.019	0.023	-0.026	0.064	795.017	0.825	0.410
interval*template	0.012	0.024	-0.034	0.058	794.994	0.527	0.599
stim*interval*template	-0.028	0.033	-0.093	0.037	795.001	-0.837	0.403

Post hoc[b]	Estimated difference	SE	lower CI	upper CI	df	z-ratio	*P* (corrected)
rest_off - draw_off	0.049	0.012	0.018	0.081	795	4.151	**<0.001**
rest_off - rest_on	0.034	0.012	0.004	0.065	795	2.996	**0.017**
rest_off - draw_on	0.047	0.012	0.016	0.078	795	4.042	**<0.001**
draw_off - rest_on	-0.015	0.012	-0.047	0.017	795	-1.245	1.000
draw_off - draw_on	-0.002	0.012	-0.034	0.030	795	-0.177	1.000
rest_on - draw_on	0.013	0.012	-0.018	0.044	795	1.091	1.000

Number of bursts[a]	Estimate	SE	lower CI	upper CI	df	t	*P*
stim	0.336	0.523	-0.687	1.357	795.516	0.642	0.521
interval	1.883	0.532	0.845	2.925	795.734	3.536	**<0.001**
template	0.085	0.511	-0.914	1.083	794.535	0.166	0.868
stim*interval	1.012	0.754	-0.464	2.484	794.943	1.341	0.180
stim*template	-0.249	0.730	-1.674	1.178	794.827	-0.341	0.733
interval*template	7.229	0.748	5.767	8.691	794.521	9.662	**<0.001**
stim*interval*template	-2.107	1.055	-4.168	-0.045	794.614	-1.997	**0.046**

Post hoc[b]	Estimated difference	SE	lower CI	upper CI	df	z-ratio	*P* (corrected)
rest_free - draw_free	-2.389	0.378	-3.388	-1.390	796	-6.322	**<0.001**
rest_free - rest_template	0.040	0.365	-0.927	1.010	796	0.108	1.000
rest_free - draw_template	-8.524	0.375	-9.515	-7.530	796	-22.758	**<0.001**
draw_free - rest_template	2.428	0.376	1.434	3.420	797	6.459	**<0.001**
draw_free - draw_template	-6.136	0.381	-7.144	-5.130	795	-16.094	**<0.001**
rest_template - draw_template	-8.564	0.372	-9.549	-7.580	797	-22.997	**<0.001**

Bursts per second[a]	Estimate	SE	lower CI	upper CI	df	t	*P*
stim	0.021	0.038	-0.054	0.096	795.692	0.537	0.592
interval	-0.095	0.039	-0.171	-0.018	795.947	-2.421	**0.016**
template	0.011	0.038	-0.063	0.084	794.538	0.282	0.778
stim*interval	0.043	0.056	-0.066	0.151	795.020	0.767	0.444
stim*template	-0.015	0.054	-0.120	0.090	794.883	-0.278	0.781
interval*template	0.047	0.055	-0.061	0.154	794.522	0.849	0.396
stim*interval*template	-0.119	0.078	-0.271	0.033	794.631	-1.534	0.126

Acceleration at burst onset[a]	Estimate	SE	lower CI	upper CI	df	t	*P*
stim	0.015	0.004	0.006	0.024	7683	3.343	**0.001**
template	0.006	0.004	-0.002	0.013	7680	1.435	0.151
interval	0.014	0.005	0.006	0.023	7671	3.174	**0.002**
stim*template	-0.015	0.005	-0.026	-0.005	7677	-2.802	**0.005**
stim*interval	-0.007	0.006	-0.019	0.005	7671	-1.132	0.257
template*interval	-0.014	0.006	-0.025	-0.003	7671	-2.535	**0.011**
stim*template*interval	0.012	0.008	-0.003	0.027	7671	1.590	0.112

controlling for burstrate, amplitude, duration during rest[a]	Estimate	SE	lower CI	upper CI	df	t	*P*
stim	0.015	0.004	0.006	0.023	7671	3.267	**0.001**
template	0.005	0.004	-0.002	0.013	7668	1.382	0.167
interval	0.014	0.005	0.006	0.023	7658	3.174	**0.002**
Burstrate_rest	0.006	0.003	0.000	0.012	7671	1.874	0.061
duration_rest_Log	-0.011	0.008	-0.027	0.004	3674	-1.451	0.147
amplitude_rest_Log	0.006	0.009	-0.011	0.024	212.400	0.710	0.478
stim*template	-0.015	0.005	-0.026	-0.004	7668	-2.755	**0.006**

stim*interval	-0.007	0.006	-0.019	0.005	7658	-1.133	0.257
template*interval	-0.014	0.006	-0.025	-0.003	7658	-2.535	**0.011**
stim*template*interval	0.012	0.008	-0.003	0.027	7658	1.628	0.104

controlling for amplitude during rest[a]	Estimate	SE	lower CI	upper CI	df	t	*P*
stim	0.015	0.004	0.006	0.024	7673	3.341	**0.001**
template	0.006	0.004	-0.002	0.014	7670	1.457	0.145
interval	0.014	0.005	0.006	0.023	7660	3.173	**0.002**
amplitude_rest_Log	0.002	0.008	-0.014	0.019	282.200	0.277	0.782
stim*template	-0.015	0.005	-0.026	-0.005	7667	-2.833	**0.005**
stim*interval	-0.007	0.006	-0.019	0.005	7660	-1.132	0.258
template*interval	-0.014	0.006	-0.025	-0.003	7660	-2.535	**0.011**
stim*template*interval	0.012	0.008	-0.003	0.027	7660	1.628	0.104

controlling for duration during rest[a]	Estimate	SE	lower CI	upper CI	df	t	*P*
stim	0.015	0.004	0.006	0.023	7673	3.296	**0.001**
template	0.005	0.004	-0.002	0.013	7670	1.358	0.174
interval	0.014	0.005	0.006	0.023	7660	3.174	**0.002**
duration_rest_Log	-0.011	0.007	-0.025	0.003	7564	-1.577	0.115
stim*template	-0.015	0.005	-0.025	-0.004	7668	-2.726	**0.006**
stim*interval	-0.007	0.006	-0.019	0.005	7660	-1.132	0.258
template*interval	-0.014	0.006	-0.025	-0.003	7660	-2.535	**0.011**
stim*template*interval	0.012	0.008	-0.003	0.027	7660	1.628	0.104

controlling for burstrate during rest[a]	Estimate	SE	lower CI	upper CI	df	t	*P*
stim	0.015	0.004	0.006	0.023	7672	3.318	**0.001**
template	0.005	0.004	-0.002	0.013	7669	1.370	0.171
interval	0.014	0.005	0.006	0.023	7660	3.174	**0.002**
Burstrate_rest	0.007	0.003	0.001	0.013	7659	2.143	**0.032**
stim*template	-0.015	0.005	-0.026	-0.004	7667	-2.776	**0.006**
stim*interval	-0.007	0.006	-0.019	0.005	7660	-1.133	0.257
template*interval	-0.014	0.006	-0.025	-0.003	7660	-2.535	**0.011**
stim*template*interval	0.012	0.008	-0.003	0.027	7660	1.628	0.104

Post hoc (interval*template)[b]	Estimated difference	SE	lower CI	upper CI	df	z-ratio	*P* (corrected)
free_post - template_post	0.002	0.003	-0.005	0.009	7682	0.711	1.000
free_post - free_pre	-0.011	0.003	-0.019	-0.003	7674	-3.527	**0.003**
free_post - template_pre	-0.001	0.003	-0.008	0.006	7682	-0.405	1.000
template_post - free_pre	-0.013	0.003	-0.020	-0.006	7682	-4.746	**<0.001**
template_post - template_pre	-0.003	0.002	-0.009	0.003	7674	-1.376	1.000
free_pre - template_pre	0.010	0.003	0.003	0.017	7682	3.630	**0.002**

Post hoc (stim*template)[b]	Estimated difference	SE	lower CI	upper CI	df	z-ratio	*P* (corrected)
free_off - template_off	0.001	0.003	-0.006	0.009	7684	0.480	1.000
free_off - free_on	-0.011	0.003	-0.020	-0.003	7681	-3.575	**0.002**
free_off - template_on	-0.001	0.003	-0.008	0.007	7684	-0.301	1.000
template_off - free_on	-0.013	0.003	-0.020	-0.006	7677	-4.735	**<0.001**
template_off - template_on	-0.002	0.002	-0.008	0.004	7683	-0.986	1.000
free_on - template_on	0.010	0.003	0.003	0.017	7683	3.947	0.001

Residual at burst onset[a]	Estimate	SE	lower CI	upper CI	df	t	*P*
stim	0.001	0.001	-0.001	0.003	5819	0.678	0.498
template	0.000	0.001	-0.002	0.002	5493	0.068	0.946
interval	0.000	0.001	-0.002	0.002	6190	0.000	1.000
stim*template	-0.001	0.001	-0.004	0.002	6146	-0.809	0.418
stim*interval	0.000	0.002	-0.003	0.003	6190	0.000	1.000
template*interval	0.000	0.001	-0.003	0.003	6190	0.000	1.000

stim*template*interval	0.000	0.002	-0.004	0.004	6190	0.000	1.000

Associatons Δaccerelation and burst amplitude[a]	Estimate	SE	lower CI	upper CI	df	t	*P*
stim	-0.003	0.005	-0.013	0.007	1070	-0.610	0.542
template	0.011	0.005	0.001	0.021	3740	2.105	**0.035**
amplitudeLog	0.001	0.018	-0.035	0.037	82	0.074	0.941
stim*amplitudeLog	-0.060	0.023	-0.105	-0.015	3616	-2.589	**0.010**
template*amplitudeLog	-0.011	0.019	-0.049	0.026	3837	-0.592	0.554
stim*template*amplitudeLog	0.062	0.026	0.010	0.114	3825	2.327	**0.020**

Associatons Δaccerelation and burst amplitude (incl. covariates)[a]	Estimate	SE	lower CI	upper CI	df	t	*P*
stim	-0.004	0.005	-0.014	0.006	1228	-0.719	0.472
template	0.011	0.005	0.000	0.021	3745	2.034	**0.042**
amplitudeLog	0.008	0.028	-0.045	0.063	342	0.306	0.760
Burstrate_rest	-0.001	0.007	-0.016	0.013	3728	-0.185	0.853
duration_rest_Log	0.024	0.018	-0.011	0.058	2051	1.358	0.175
amplitude_rest_Log	-0.007	0.024	-0.053	0.039	393	-0.291	0.771
stim*amplitudeLog	-0.060	0.023	-0.106	-0.015	3621	-2.622	**0.009**
template*amplitudeLog	-0.012	0.019	-0.050	0.025	3819	-0.628	0.530
stim*template*amplitudeLog	0.061	0.026	0.009	0.113	3801	2.297	**0.022**

Bold numbers indicate significant effects. SE = standard error; CI = Confidence interval; df = degrees of freedom; t = t-score; *P* = *P*-value; *P* (corr) = Bonferroni-corrected *P*-value.

[a] two-sided linear mixed-effects model

[b] two-sided pairwise estimated marginal means post hoc test (Bonferroni corrected for multiple comparisons)

References

1 San Luciano, M. *et al.* Digitized Spiral Drawing: A Possible Biomarker for Early Parkinson's Disease. *PloS one* **11**, e0162799, doi:10.1371/journal.pone.0162799 (2016).
