# Supplementary material for: Subthalamic stimulation modulates context-dependent effects of beta bursts during fine motor control
Source: Nat Commun. 2024 Apr 12;15:3166. doi: 10.1038/s41467-024-47555-3 (PMC11009405; doi:10.1038/s41467-024-47555-3)
Supplement: Supplementary file 3 — Reporting Summary [file 41467_2024_47555_MOESM3_ESM.pdf]

nature portfolio

Corresponding author(s): Sergiu Groppa

Last updated by author(s): Feb 29, 2024

Reporting Summary

Nature Portfolio wishes to improve the reproducibility of the work that we publish. This form provides structure for consistency and transparency in reporting. For further information on Nature Portfolio policies, see our Editorial Policies and the Editorial Policy Checklist.

Statistics

For all statistical analyses, confirm that the following items are present in the figure legend, table legend, main text, or Methods section.

n/a	Confirmed	
☐	☒	The exact sample size (*n*) for each experimental group/condition, given as a discrete number and unit of measurement
☐	☒	A statement on whether measurements were taken from distinct samples or whether the same sample was measured repeatedly
☐	☒	The statistical test(s) used AND whether they are one- or two-sided<br>*Only common tests should be described solely by name; describe more complex techniques in the Methods section.*
☐	☒	A description of all covariates tested
☐	☒	A description of any assumptions or corrections, such as tests of normality and adjustment for multiple comparisons
☐	☒	A full description of the statistical parameters including central tendency (e.g. means) or other basic estimates (e.g. regression coefficient) AND variation (e.g. standard deviation) or associated estimates of uncertainty (e.g. confidence intervals)
☐	☒	For null hypothesis testing, the test statistic (e.g. *F*, *t*, *r*) with confidence intervals, effect sizes, degrees of freedom and *P* value noted<br>*Give P values as exact values whenever suitable.*
☒	☐	For Bayesian analysis, information on the choice of priors and Markov chain Monte Carlo settings
☐	☒	For hierarchical and complex designs, identification of the appropriate level for tests and full reporting of outcomes
☐	☒	Estimates of effect sizes (e.g. Cohen's *d*, Pearson's *r*), indicating how they were calculated

Our web collection on statistics for biologists contains articles on many of the points above.

Software and code

Policy information about availability of computer code

Data collection	Neurophysiological data were collected on a TMSi porti device (TMS International, Enschede, The Netherlands) using TMSi software and drivers (version 7_2_144). Spiral data were collected on a digital graphics tablet (Wacom Intuos Pro – Creative Pen Tablet, size L, resolution = 5080 lpi, maximal sample rate 200 Hz, pressure sensitivity = 2048 levels, Wacom Technology Corporation, Vancouver, WA) using the software Neuroglyphics (version Oct 1, 2018, http://www.neuroglyphics.org/)
Data analysis	Spiral data were preprocessed with the kinematics toolbox (version V.1.0, http://www.diedrichsenlab.org/toolboxes/toolbox_kinematics.htm) and custom Matlab-code (The MathWorks, version R2017a).<br>The analysis of local field potential data was performed in Spike2 (Cambridge Electronic Design, version 8.10), FieldTrip36 (version 20220310, https://www.fieldtriptoolbox.org/), and the timeWarp function (version 2006) from EEGLab (https://sccn.ucsd.edu/eeglab/index.php) as implemented in Matlab. Beta bursts and their characteristics were analysed using custom Matlab-code (available on https://github.com/manubange/SpiralBeta).<br>Electrode positions were modelled by Lead-DBS (Version 2.5.2, https://www.lead-dbs.org/)<br>Statistical analyses were performed using linear mixed effects regression models implemented in R-Studio (version 2022.02.1, http://www.rstudio.com/) in the lme4-package (version 1.1-32, https://cran.r-project.org/web/packages/lme4/index.html). Illustrations were created with the R ggplot2 package (version 3.4.0, https://ggplot2.tidyverse.org), Fieldtrip, or Matlab.

For manuscripts utilizing custom algorithms or software that are central to the research but not yet described in published literature, software must be made available to editors and reviewers. We strongly encourage code deposition in a community repository (e.g. GitHub). See the Nature Portfolio guidelines for submitting code & software for further information.

Data

Policy information about availability of data

All manuscripts must include a data availability statement. This statement should provide the following information, where applicable:

- Accession codes, unique identifiers, or web links for publicly available datasets
- A description of any restrictions on data availability
- For clinical datasets or third party data, please ensure that the statement adheres to our policy

The data that support the findings of this study are available upon request from the corresponding author. Participant consent allows sharing the data exclusively for scientific purposes, thus we cannot openly deposit the full original dataset online. A minimum example dataset (including scripts) is available on https://github.com/manubange/SpiralBeta.git (DOI: 10.5281/zenodo.10795009)68. Source data are provided with this paper.

Research involving human participants, their data, or biological material

Policy information about studies with human participants or human data. See also policy information about sex, gender (identity/presentation), and sexual orientation and race, ethnicity and racism.

Reporting on sex and gender	Participants of both biological sexes were included (15 male and 4 female patients). No consent has been obtained for reporting individual level data. Because the aim of the study is to investigate the role of basal ganglia beta oscillations for fine controlled motor skills in Parkinson's disease and because the sample size is to low to quantify potential sex differences we did not conduct additional sex- and gender-based analyses. In the source data file, the data are shown excluding information on sex or gender.
Reporting on race, ethnicity, or other socially relevant groupings	Data of this study included residents of the Federal Republik of Germany.
Population characteristics	Patients' age was 67.68 (range: 49-80, standard deviation: 7.25). Handedness was assessed by self-report (1 left handed person).
Recruitment	19 patients with Parkinson's Disease who had undergone subthalamic (STN) deep brain stimulation (DBS) surgery prior to the experimental recordings were recruited at the University Medical Center of the Johannes Gutenberg University Mainz. The indication for DBS treatment was made purely on clinical grounds, irrespective of the study participation. Patients were asked if they were interested in participating in the study prior to the surgery. Participation did not affect the clinical treatment, making self-selection bias less likely.
Ethics oversight	The study was approved by the local ethics committee (State Medical Association of Rhineland-Palatinate)

Note that full information on the approval of the study protocol must also be provided in the manuscript.

Field-specific reporting

Please select the one below that is the best fit for your research. If you are not sure, read the appropriate sections before making your selection.

☒ Life sciences ☐ Behavioural & social sciences ☐ Ecological, evolutionary & environmental sciences

For a reference copy of the document with all sections, see nature.com/documents/nr-reporting-summary-flat.pdf

Life sciences study design

All studies must disclose on these points even when the disclosure is negative.

Sample size	Due to the invasive nature of subthalamic nucleus (STN) recordings and deep brain stimulation (DBS) were were not able to record pilot data for computing the effect size of STN local field potential (LFP) changes related to drawing movements. However, given the very good signal-to-noise ratio of invasive STN LFP recordings with a typical sample size of 10-15 (see refs 21, 24, 26 and 31 in the article) we considered a slightly increased sample size appropriate.
Data exclusions	LFP data that could not be unambiguously assigned to the corresponding trials were excluded from the LFP analysis.
Replication	The main findings that DBS speeds up drawing, reduces the burst amplitude, and affects the association between beta bursts and the acceleration during free drawing were replicated once when excluding inaccurate trials and once when excluding seven tremor dominant patients.
Randomization	We performed a test stimulation before the experiment to determine the optimal stimulation currents that provided the best clinical benefit without any side effects. Because one researcher controlled recording and stimulation while another researcher assessed clinical scores and performed the experimental procedures with the patients, we were able to pseudo-randomize and counterbalance the order of the stimulation condition.

Blinding	We performed a test stimulation before the experiment to determine the optimal stimulation currents that provided the best clinical benefit without any side effects. One researcher controlled recording and stimulation while another researcher assessed clinical scores and performed the experimental procedures with the patients in pseudo-randomized order. Neither the performing researcher, nor the patients were told which stimulation condition was currently performed, thus both were blinded to the respective condition. The researcher recording and controlling the stimulation, however, could not undergo blinding because he was responsible for actively switching the stimulation on or off.

Reporting for specific materials, systems and methods

We require information from authors about some types of materials, experimental systems and methods used in many studies. Here, indicate whether each material, system or method listed is relevant to your study. If you are not sure if a list item applies to your research, read the appropriate section before selecting a response.

Materials & experimental systems

n/a	Involved in the study
☒	☐ Antibodies
☒	☐ Eukaryotic cell lines
☒	☐ Palaeontology and archaeology
☒	☐ Animals and other organisms
☒	☐ Clinical data
☒	☐ Dual use research of concern
☒	☐ Plants

Methods

n/a	Involved in the study
☒	☐ ChIP-seq
☒	☐ Flow cytometry
☐	☒ MRI-based neuroimaging

Magnetic resonance imaging

Experimental design

Design type	Not applicable. We only used structural MRI (T1 Sequence) for DBS electrode reconstruction
Design specifications	Not applicable.
Behavioral performance measures	Not applicable.

Acquisition

Imaging type(s)	Structural MRI
Field strength	3.0 T
Sequence & imaging parameters	T1 mprage, TE 2.52, TR 1900, TI 900, Slice thickness 1mm, FOV 256x256, Flip Angle 9 (Siemens Trio)
Area of acquisition	a whole brain scan was used

Diffusion MRI ☐ Used ☒ Not used

Preprocessing

Preprocessing software	We used the advanced electrode localization pipeline (in Lead-DBS version 2.5.2) with default settings for electrode reconstruction
Normalization	The pre-operative MRI scan (T1 sequence) was linearly co-registered with the postoperative anatomical CT and nonlinearly warped to the MNI template (ICBM 2009bNonlinear Asymmetric) using the Advanced Normalization Tools (ANTs)
Normalization template	ICBM 2009bNonlinear Asymmetric
Noise and artifact removal	*Describe your procedure(s) for artifact and structured noise removal, specifying motion parameters, tissue signals and physiological signals (heart rate, respiration).*
Volume censoring	Not applicable

Statistical modeling & inference

Model type and settings	Not applicable
Effect(s) tested	Not applicable

Specify type of analysis: ☐ Whole brain ☐ ROI-based ☐ Both

Statistic type for inference (See Eklund et al. 2016)	Not applicable
Correction	Not applicable

Models & analysis

n/a	Involved in the study
☒	☐ Functional and/or effective connectivity
☒	☐ Graph analysis
☒	☐ Multivariate modeling or predictive analysis
